# Optogenetic current in myofibroblasts acutely alters electrophysiology and conduction of co-cultured cardiomyocytes

**DOI:** 10.1038/s41598-021-83398-4

**Published:** 2021-02-24

**Authors:** Geran M. Kostecki, Yu Shi, Christopher S. Chen, Daniel H. Reich, Emilia Entcheva, Leslie Tung

**Affiliations:** 1 Department of Biomedical Engineering, Johns Hopkins University, 720 Rutland Ave., Baltimore, MD 21205 USA; 2 Department of Physics and Astronomy, Johns Hopkins University, Baltimore, MD 21218 USA; 3 Biological Design Center, Department of Biomedical Engineering, Boston University, Boston, MA USA; 4 Wyss Institute for Biologically Inspired Engineering, Harvard University, Boston, MA USA; 5 Department of Biomedical Engineering, George Washington University, Washington, DC USA

**Keywords:** Optogenetics, Arrhythmias, Computer modelling, Multicellular systems, Myocardial infarction

## Abstract

Interactions between cardiac myofibroblasts and myocytes may slow conduction and generate spontaneous beating in fibrosis, increasing the chance of life-threatening arrhythmia. While co-culture studies have shown that myofibroblasts can affect cardiomyocyte electrophysiology in vitro, the extent of myofibroblast-myocyte electrical conductance in a syncytium is unknown. In this neonatal rat study, cardiac myofibroblasts were transduced with Channelrhodopsin-2, which allowed acute and selective increase of myofibroblast current, and plated on top of cardiomyocytes. Optical mapping revealed significantly decreased conduction velocity (− 27 ± 6%, p < 10^–3^), upstroke rate (− 13 ± 4%, p = 0.002), and action potential duration (− 14 ± 7%, p = 0.004) in co-cultures when 0.017 mW/mm^2^ light was applied, as well as focal spontaneous beating in 6/7 samples and a decreased cycle length (− 36 ± 18%, p = 0.002) at 0.057 mW/mm^2^ light. In silico modeling of the experiments reproduced the experimental findings and suggested the light levels used in experiments produced excess current similar in magnitude to endogenous myofibroblast current. Fitting the model to experimental data predicted a tissue-level electrical conductance across the 3-D interface between myofibroblasts and cardiomyocytes of ~ 5 nS/cardiomyocyte, and showed how increased myofibroblast-myocyte conductance, increased myofibroblast/myocyte capacitance ratio, and increased myofibroblast current, which occur in fibrosis, can work in tandem to produce pro-arrhythmic increases in conduction and spontaneous beating.

## Introduction

Myocardial injury or stress (e.g., due to myocardial infarction, pressure/volume overload, aging, or myocarditis) causes release of paracrine factors, including transforming growth factor-β1 (TGF-β1), which causes fibroblasts to differentiate into myofibroblasts (MFBs), initiating fibrosis^1–5^. MFBs express α-smooth muscle actin (α-SMA) fibers and contract, which stabilizes and shrinks the injured area. They also secrete extracellular matrix which replaces dead cells and further mechanically stabilizes the tissue^1–5^.

Along with these changes, arrhythmia risk is significantly increased^6,7^. There are many contributing factors to this, such as increased heterogeneity of cardiomyocyte (CM) coupling causing zigzag propagation or electrical block, as well as ion channel remodeling in CMs themselves^3,6–8^. However, an additional factor is the effect of MFBs themselves on CM electrophysiology^6,7^, since MFBs can remain in a differentiated state in the injured area years after injury^9^. In vitro, the addition of MFBs to CMs slows CM conduction velocity (CV), and increases spontaneous beating rate^6,7^, so these effects may contribute to the arrhythmia observed in vivo. It is believed that such effects are due to electrical coupling between CMs and less electrically polarized MFBs which causes current to flow into CMs at rest, thereby raising CM resting or maximum diastolic potential (MDP), which can then inactivate sodium channels or generate spontaneous activity^6,7^. These events can contribute to the occurrence^10^ and complexity^11^ of spiral waves that have been observed with an increasing fraction of MFBs in co-culture. However, such pro-arrhythmic MFB-CM interactions may also be caused by paracrine and mechanical mechanisms^12^. Furthermore, the extent of electrical coupling between MFBs and CMs has only been measured between cell pairs^13,14^, which differs from the situation in vivo in that the cells are sparse and interact along a narrow interface.

In this study, MFBs were plated on top of CMs, so that their interactions with CMs occurred over a large area in 3-D, and were transduced with Channelrhodopsin-2 (ChR2), a relatively non-selective cation channel that opens in response to light^15^, to acutely depolarize them. This MFB-specific perturbation enabled the study of acute effects of MFB depolarizing current on the macroscopic electrophysiological properties of syncytia containing co-cultured MFBs and CMs, and was used in tandem with computational modeling to estimate MFB-CM electrical conductance. The model was then used to better understand the mechanism of these effects and to show how changes in MFB-CM conductance, ratio of MFB to CM capacitance, and level of endogenous MFB currents may increase arrhythmia in fibrosis. These results have been reported in large part in a preprint of this work^16^.

## Results

### Co-culture of cardiomyocytes with ChR2-transduced myofibroblasts

**Figure 1:**
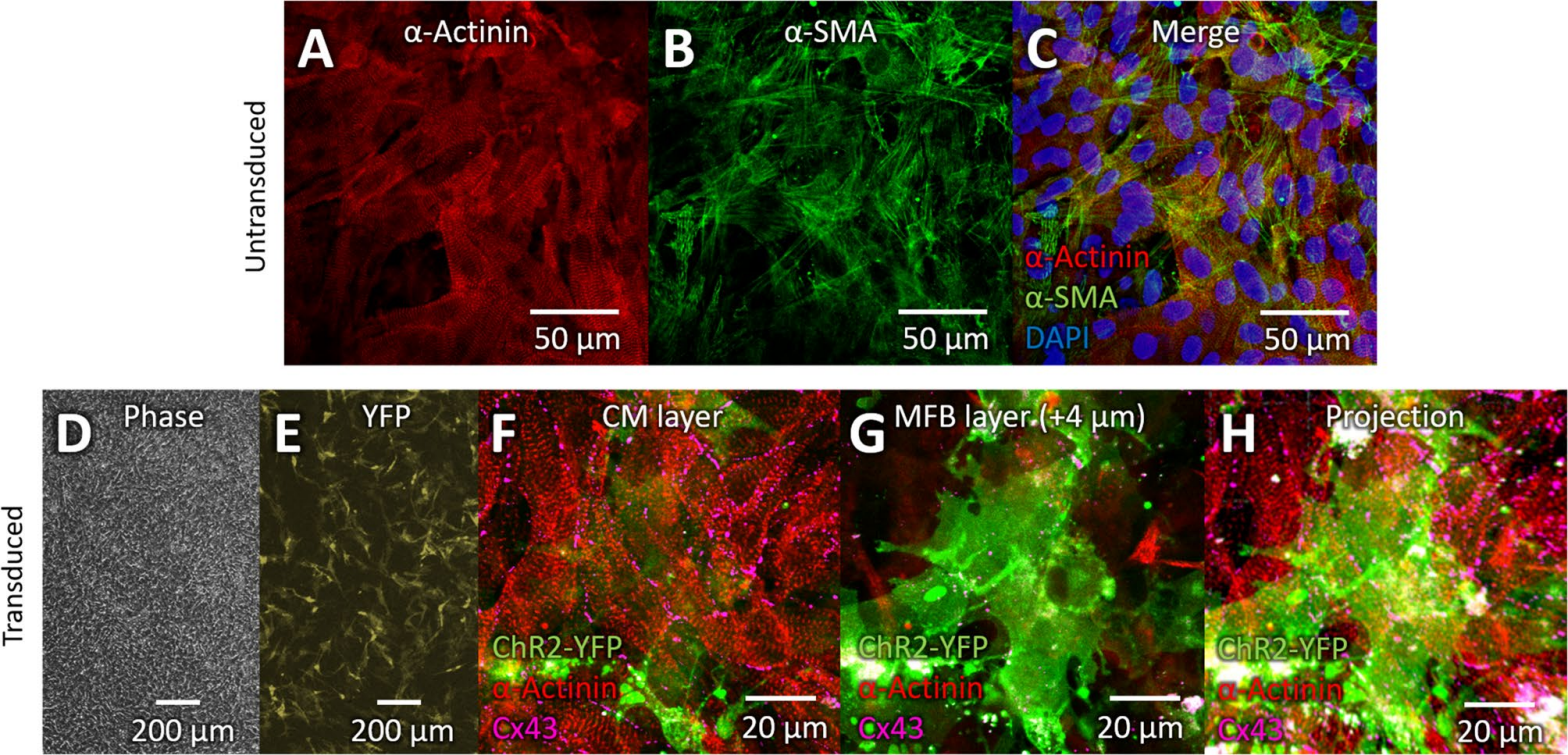
Co-culture of cardiomyocytes and ChR2-transduced myofibroblasts. (**A**–**C)** Confocal image of co-culture of MFBs and CMs. α-actinin (**A**, red) marks CMs, and α-smooth muscle actin (**B**, α-SMA, green) marks MFBs. (**C)** Merge of (**A)** and (**B)** with DAPI (blue) to stain nuclei. (**D)** Phase-contrast image of CMs co-cultured with ChR2-MFBs. (**E**) Fluorescence image of same sample and area as in D, with YFP marking transduced MFBs. (**F–H**) Confocal images from 18 μm-thick z-stack of transduced MFBs and CMs. ChR2-YFP (green) marks transduced MFBs, α-actinin (red) marks CMs, and violet shows connexin43 (Cx43). ( **F**) CM layer of z-stack showing gap junctions between CMs. (**G**) Image from 4 μm above F showing MFBs on top of CMs, as well as Cx43 puncta, apparently between CMs and MFBs. (**H**) Maximum intensity projection of entire z-stack. Each layer of the z-stack is shown in Supplementary Fig. 1.

To assess whether inward current in MFBs can alter CM electrophysiology at a tissue level, neonatal rat cardiac fibroblasts were transduced with ChR2 and differentiated into MFBs by treatment with TGF-β1, then plated on top of neonatal rat ventricular CM monolayers. Confocal imaging of MFBs with CMs demonstrated continued expression of α-SMA by MFBs two days after plating on CMs and concomitant cessation of TGF-β1 treatment (Fig. 1A–C). Wide-field (~ 2 mm) imaging of co-cultures of ChR2-transduced MFBs (ChR2-MFBs) showed that they formed a homogeneous, dense network over a wide area of CMs (Fig. 1D) and continued to express ChR2 during co-culture (Fig. 1E). Confocal imaging showed confluent CMs (Fig. 1F) with ChR2-MFBs resting on top of them (Fig. 1G and H; see Supplementary Fig. 1 for full z-stack), as well as Cx43 puncta in the MFB cell layer (Fig. 1G), suggesting expression of Cx43 by MFBs.

### Myofibroblast current can cause electrophysiological changes in cardiomyocytes

**Figure 2:**
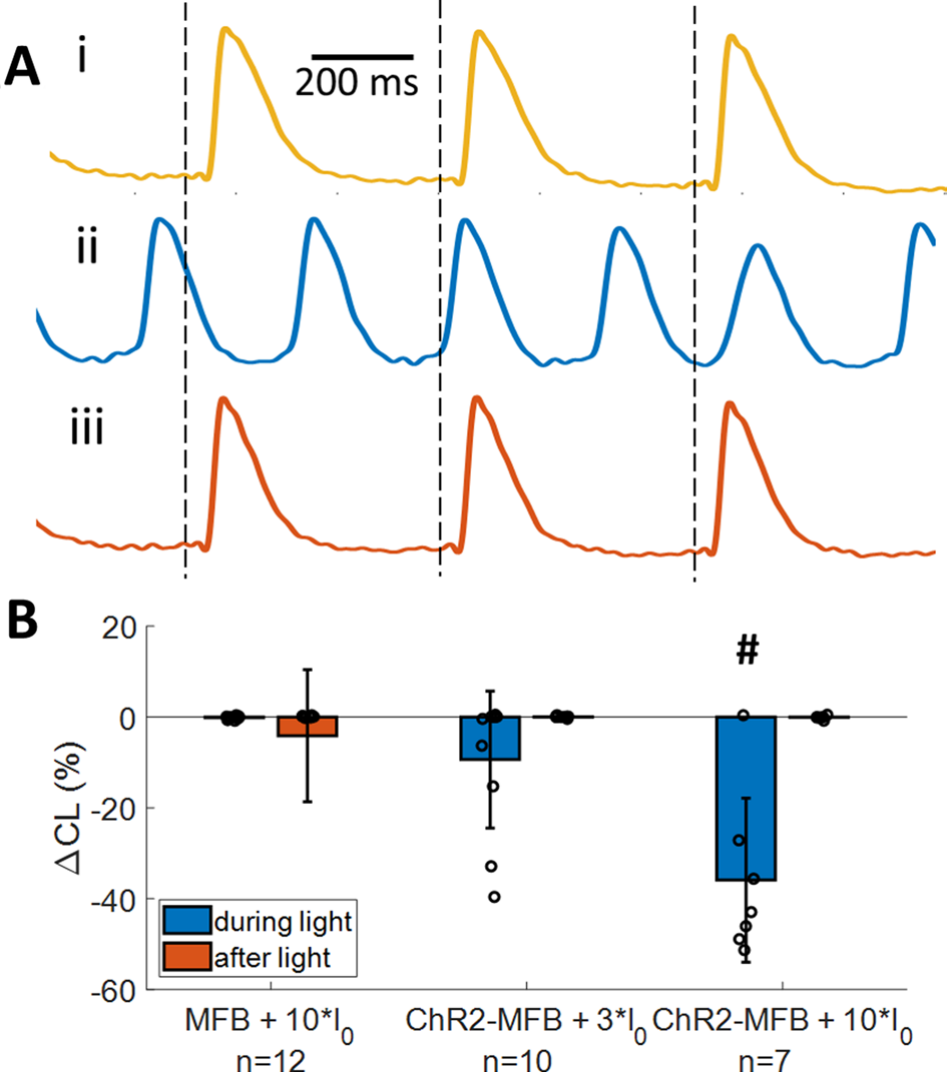
Inward current in myofibroblasts causes spontaneous beating in co-cultured cardiomyocyte syncytia. (**A**) Averaged voltage traces of a co-culture of ChR2-transduced MFBs (ChR2-MFBs) with CMs before (i, gold), during (ii, blue), and after (iii, orange) application of 10*I_0_ blue light (I_0_ = 0.0057 mW/mm^2^, the lowest light intensity at which functional effects were generally observed) to activate ChR2 current in MFBs. Vertical dashed lines show time of pacing. Activation maps are shown in Supplementary Fig. 2. (**B**) Percent change (from value prior to light application) in cycle length (CL) during and after application of light at different power levels during 500 ms CL pacing, for co-cultures of CMs with MFBs or ChR2-MFBs. 4/10 samples beat spontaneously at 3*I_0_, while 6/7 beat spontaneously at 10*I_0_. # indicates p < 0.005 between ChR2-MFB and MFB co-culture responses during light.

In ChR2-MFB co-cultures with CMs, electrically paced at 500 ms cycle length (CL, Fig. 2Ai), application of continuous blue light to open ChR2 channels could induce diastolic depolarization and spontaneous beating at a rate faster than the 500 ms paced CL (Fig. 2Aii), whereas this did not occur in MFB co-cultures with CMs. Cessation of light (and therefore ChR2 current) caused spontaneous beating to stop (Fig. 2Aiii). This spontaneous beating in response to light occurred in ChR2-MFB co-cultures, but in none of the MFB co-cultures (Fig. 2B), thus precluding thermal or other non-specific effects of the applied light. Data across multiple samples showed that 3*I_0_ light (I_0_ = 0.0057 mW/mm^2^, approximately the lowest light level at which effects could be seen) caused spontaneous beating faster than the 500 ms paced CL in 4/10 (four of ten) samples, and that beating CL decreased as ChR2 current increased (Fig. 2B). Under 10*I_0_ light, 6/7 co-cultures beat spontaneously, and the change in CL was − 36 ± 18% from a paced baseline of 500 ± 1 ms (n = 7; p = 0.002 vs. MFB co-cultures, Fig. 2B). Activation maps showed spontaneous beating was focal and in 5/6 cases originated in a location different than the pacing site (See Supplementary Fig. 2 for example).

**Figure 3:**
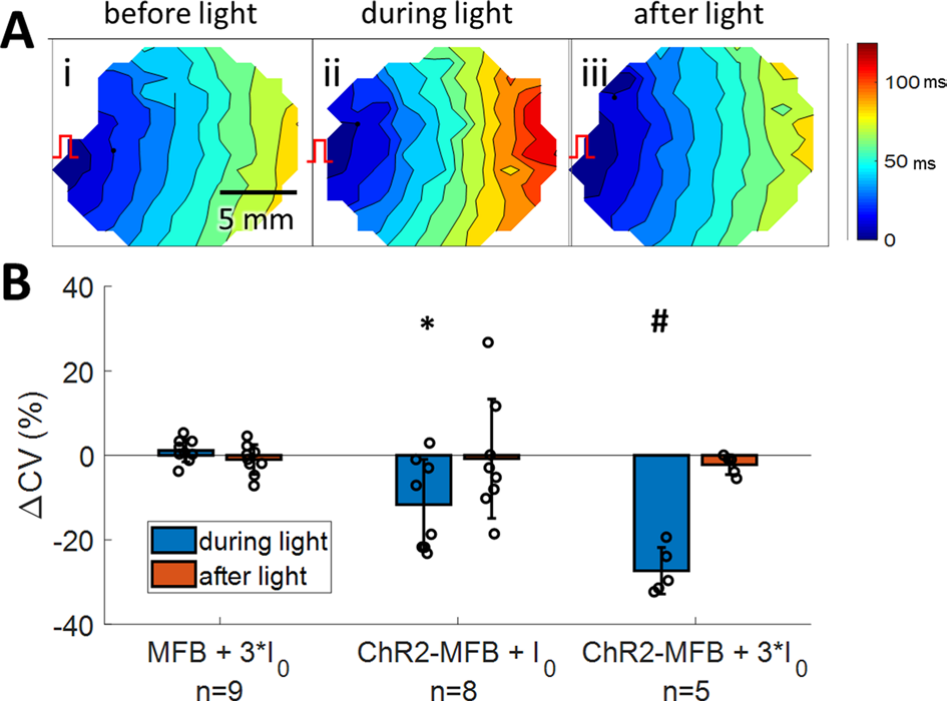
Inward current in myofibroblasts causes slowing in co-cultured cardiomyocyte syncytia. (**A**) Activation maps of a co-culture of ChR2-MFBs with CMs before (**i**), during (**ii**), and after (**iii**) application of 3*I_0_ blue light to activate ChR2 current in MFBs. Color bar at right shows activation time scale. Isochrones are 10 ms apart. Red pacing marker illustrates location of pacing. (**B**) Percent change (from value prior to light application) in conduction velocity (CV) during and after application of light at different power levels during 500 ms CL pacing, for co-cultures of CMs with MFBs or ChR2-MFBs. Data for 10*I_0_ is not shown since almost all samples beat spontaneously with CL less than 500 ms at this intensity. * indicates p < 0.05, # indicates p < 0.005 between ChR2-MFB and MFB co-culture responses during light.

Addition of ChR2-MFBs trended towards reducing CV relative to CM-only cultures during 500 ms CL pacing, although not at the level of significance (17.7 ± 5.3, n = 8 vs. 20.9 ± 4.3, n = 14; p = 0.17). For ChR2-MFB co-cultures (Fig. 3Ai), application of light slowed CV (Fig. 3Aii), and this slowing was reversed when the light was turned off (Fig. 3Aiii). CV decreased further as light intensity was increased, until the onset of spontaneous beating prevented further comparison of CVs (since CV can change from beating rate changes alone) (Fig. 3B). There was significant slowing in ChR2-MFB co-cultures at light levels as low as I_0_ (ΔCV = –‍12 ± 11% from a baseline of 17.7 ± 5.3 cm/s, n = 8; p = 0.01 vs. MFB co-cultures). Slowing was even greater at 3*I_0_ (ΔCV = –27 ± 5% from a baseline of 14.8 ± 3.6 cm/s, n = 5; p < 10^–3^ vs. MFB co-cultures, Fig. 3B).

**Figure 4:**
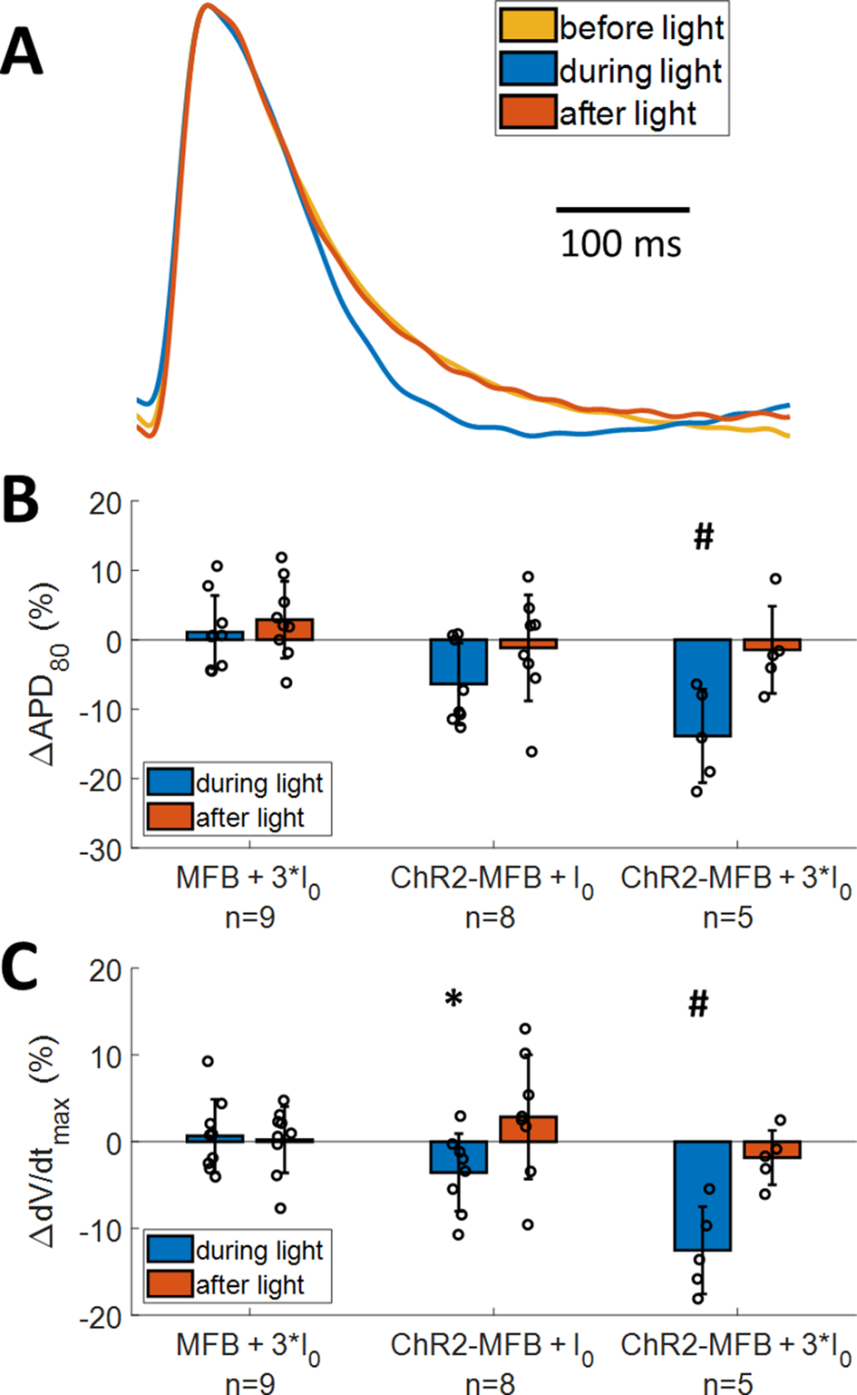
Inward current in myofibroblasts changes action potential (AP) characteristics in co-cultured myofibroblast syncytia. (**A**) Averaged AP trace before (gold), during (blue), and after (orange) application of 3*I_0_ blue light to activate ChR2 current in ChR2-MFBs. (**B-C**) Percent change (from value prior to light application) in action potential duration (APD_80_, **B**) and maximal upstroke rate (dV/dt, **C**) during and after application of light at different power levels during 500 ms CL pacing, for co-cultures of CMs with MFBs or ChR2-MFBs. * indicates p < 0.05, # indicates p < 0.005 between ChR2-MFB and MFB co-culture responses during light.

Addition of ChR2-MFBs to CM cultures significantly reduced action potential duration at 80% repolarization (APD_80_, from 191 ± 22, n = 14 to 165 ± 28 ms, n = 8; p = 0.03). Light-induced inward current further decreased APD_80_, which reversed upon removal of light (Fig. 4A). Across multiple samples, application of 3*I_0_ light resulted in a significant decrease in APD_80_ in ChR2-MFB co-cultures (− 14 ± 7%, n = 5; p = 0.004 vs. MFB co-cultures, Fig. 4B) and significantly decreased upstroke rate (− 13 ± 4%, n = 5, from a baseline of 3.8 ± 0.4%/ms; p = 0.002 vs. MFB co-cultures, Fig. 4C). P-‍values for the experimentally measured values calculated using other methods (paired and equal variance, instead of unequal variance t-test) are provided in Supplementary Table 1.

Acute application of a ChR2-saturating level of light (1.2 mW/mm^2^, equal to 210*I_0_) to ChR2-MFBs did not affect MFB contractility and force generation, as measured for single cells seeded on flexible micropost arrays (Supplementary Fig. 3), excluding the possibility that inward current in MFBs caused them to contract and potentially influence CMs by activating mechanosensitive channels.

### Insights from a mathematical model of co-cultures of cardiomyocytes and ChR2-transduced myofibroblasts

**Figure 5:**
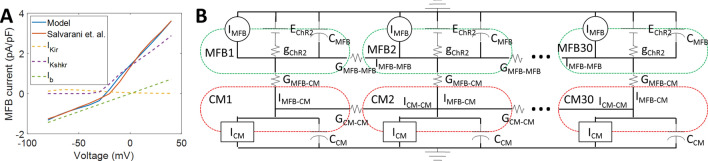
Computational model. (**A**) Endogenous cardiac MFB currents were modeled by adjusting the current conductances in the Sachse et al. fibroblast model^34^ to match the current–voltage relationship found by Salvarani et al.^13^ for TGF-β1-treated MFBs. The dashed lines show the contributions of individual currents. (**B**) The ChR2 channel model (*g*_ChR2_, *E*_ChR2_) from Williams et al.^33^ was added to endogenous MFB currents (*I*_MFB_ in figure, *I*_MFB,endo_ in equations). MFBs were electrically connected (*G*_MFB-CM_) to neonatal rat cardiomyocytes (CMs) as modeled by Korhonen et al.^17^ which were connected to each other (*G*_CM-CM_) to form a 30-cell, 1.5 mm 1-D cable. MFBs were only connected to each other (*G*_MFB-MFB_ = *G*_CM-CM_) in Supplementary Fig. 5, otherwise *G*_MFB-MFB_ = 0. Dashed lines show outer boundaries of CMs (red) and MFBs (green). Formulas for variables and values for parameters are listed in Supplementary Tables 2–4.

To better understand these results and use them to estimate MFB-CM electrical conductance, *G*_MFB-CM_, in a syncytium, which is very difficult to measure experimentally, a numerical model of a cable of 30 neonatal rat ventricular CMs connected to MFBs transduced with ChR2 current was created (Fig. 5, Supplementary Tables 2–4). Modifications were made to the neonatal rat Korhonen model^17^ to allow for stimulation at 500 ms CL in the cable, as well as to match experimental data (see Supplementary Tables 2–7 for details). The changes lowered resting potential from − 67 to − 77 mV, which is similar to other published data^13,18,19^. Minimum capture CL decreased from 420 to 200 ms (− 80 pA/pF, 0.5 ms stimulation), which is similar to what we have reported previously (~ 250 ms^20^). CV increased from 4.5 to 21.0 cm/s, the same as the experimentally measured CV (20.9 ± 4.3 cm/s). APD_80_ was also reduced from 208 to 181 ms (Supplementary Fig. 4 and Supplementary Table 8), more closely matching our measured APD_80_ of 191 ± 22 ms. While the addition of ChR2-MFBs to the CM cable caused 2:1 block at 500 ms CL in the original Korhonen model, ChR2-MFB/CM cables using the modified model could be stimulated at 500 ms CL, and had a CV and APD_80_ of 17.5 cm/s and 173 ms, respectively, similar to our measured values of 17.7 ± 5.3 cm/s and 165 ± 28 ms (Supplementary Table 8). Neighboring MFBs were not connected to each other to prevent them from having “double-sided” effects on CMs^21^ by creating an alternate current path. However, additional modeling (with MFB-MFB conductance = CM-CM conductance) showed that this alternate current path had little effect on the results (Supplementary Fig. 5).

**Figure 6:**
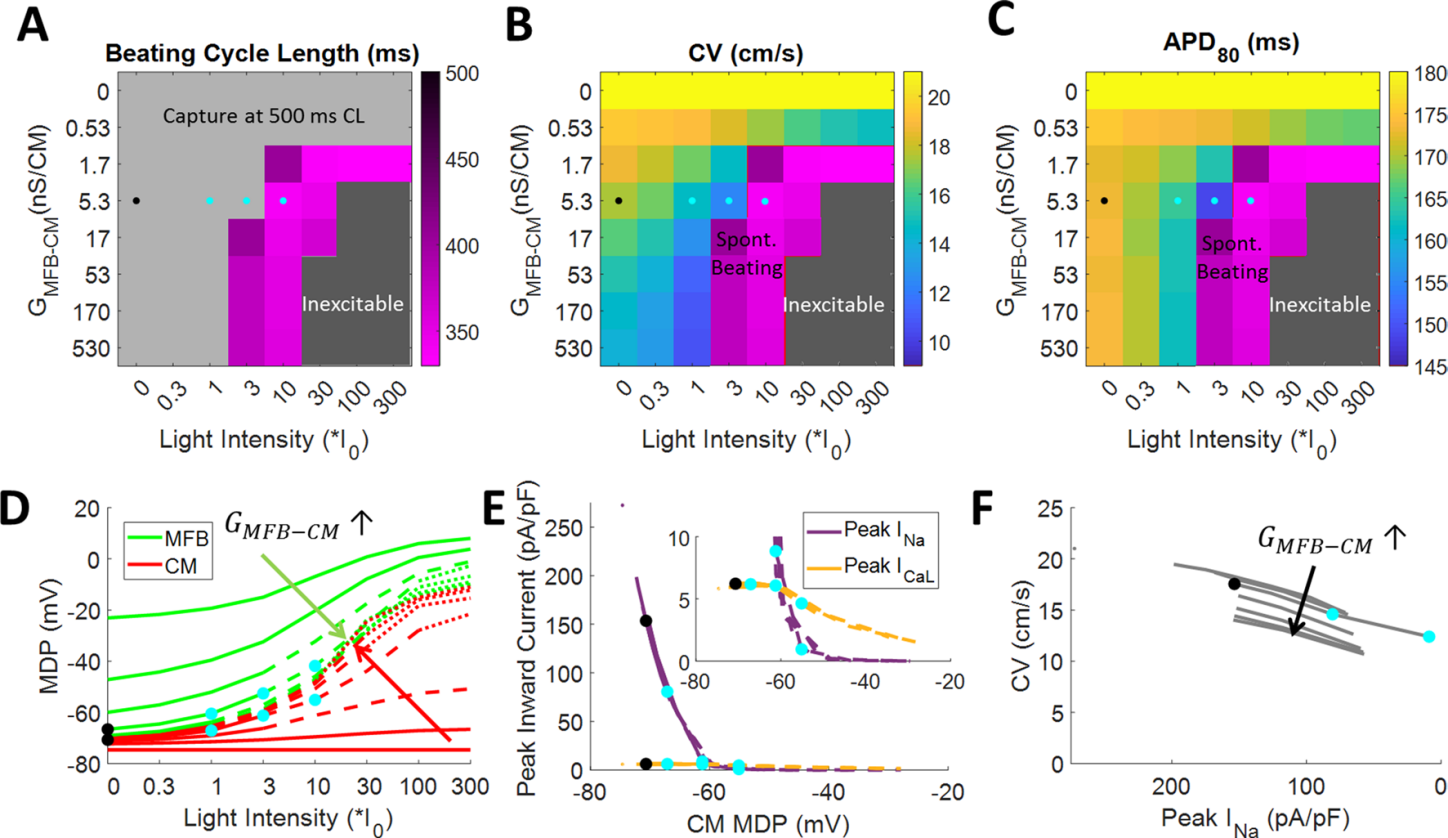
Modeling results of myofibroblasts co-cultured with cardiomyocytes. A cable of 30 pairs of MFBs and CMs in the same 0.4:1 cell ratio used in experiments were paced at 500 ms CL. Color maps show beating cycle length (**A**), conduction velocity (**B**), and APD_80_ (**C**) at different MFB-CM conductances (*G*_MFB-CM_) and light intensities. MFB-CM conductance is varied in half-log_10_ increments around the estimated MFB-CM conductance (5.3 nS/CM). Gray region in A denotes that spontaneous beating, if present, was slower than the 500 ms paced CL. CV and APD_80_ were not calculated for the purple region in B and C since spontaneous beating prevented capture at 500 ms CL pacing. Dark gray region denotes that cells were inexcitable. Black dots indicate modeled values without light, and blue dots indicate modeled values at light power levels used in experiments, all at the estimated MFB-CM conductance of 5.3 nS/CM. (**D**) Maximum diastolic potential (MDP) of MFBs (green) and CMs (red). (**E**) Peak inward sodium current (purple) and L-type calcium current (gold) versus CM maximum diastolic potential. Inset shows same data with an expanded scale. (**F**) Conduction velocity versus peak sodium current. (**D–F**) MFB-CM conductance starts at 0 then increases in half-log_10_ increments from 5.3*10^–1^ nS/CM to 5.3*10^2^ nS/CM. Solid lines show conditions that allowed capture at the 500 ms paced CL. Dashed lines show conditions that caused spontaneous beating in excess of the pacing rate. Dotted lines show conditions that caused cells to be inexcitable. Black dots indicate modeled values without light and blue dots indicate modelled values at light power levels used in experiments, all at the estimated MFB-CM conductance of 5.3 nS/CM.

Using our model, a broad parameter space was explored, where *G*_MFB-CM_ and light intensity were varied. The model showed that at light levels < 3*I_0_ or *G*_MFB-CM_ < 1.7 nS/CM, no spontaneous beating was produced, since either there was too little ChR2 current produced, or this current was unable to depolarize CMs, respectively (Fig. 6A and Supplementary Fig. 6A). However, at *G*_MFB-CM_ = 5.3 nS/CM (the value for which the model best fit our experimental data), spontaneous beating could exceed the pacing rate (500 ms CL) in response to a light level > 3*I_0_ (Fig. 6A and Supplementary Fig. 6A). This behavior agreed with our experiments, in which 4/10 of our samples beat spontaneously at 3*I_0_, while 6/7 samples beat faster than the paced rate when stimulated with 10*I_0_. This higher level of light reduced CL by 30% in the model, similar to our experiments in which CL was reduced by 36 ± 18% (Supplementary Figs. 7 and 8A). Furthermore, the model predicted that CMs become inexcitable at high light levels and high *G*_MFB-CM_ (Fig. 6A, dark gray area), but we were unable to record transmembrane voltage at such high light levels due to optical crosstalk.

The model also showed conduction slowing with addition of MFBs, and that this slowing increased with increasing *G*_MFB-CM_ or light intensity (Fig. 6B). With *G*_MFB-CM_ = 5.3 nS/CM, addition of MFBs decreased CV by 3.5 cm/s (Fig. 6B, compare box marked by black dot to top row, where *G*_MFB-CM_ = 0, which is equivalent to CMs-only in the model), similar to the 3.2 cm/s found experimentally (Supplementary Table 8). Application of I_0_ and 3*I_0_ light decreased CV by 17% and 29%, respectively (Fig. 6B, compare CV values in boxes marked by blue dots to the box marked by the black dot), similar to experiments (− 12 ± 11% and − 27 ± 5%, respectively, Supplementary Fig. 8B), while higher levels of light (blue dot at 10*I_0_) caused loss of capture due to spontaneous beating, as discussed for Fig. 6A.

Addition of MFBs to CMs decreased APD_80_ by 8 ms (Fig. 6C, black dot vs. top row, which is equivalent to the absence of MFBs), somewhat less than the decrease found experimentally (26 ms, Supplementary Table 8). Application of I_0_ and 3*I_0_ light decreased APD_80_ by 5% and 14%, respectively (Fig. 6C, compare blue dots with black dot), matching the experimental results (− 6 ± 6% and − 14 ± 7%, Supplementary Fig. 8C). Further investigation showed that the decrease in APD_80_ in response to light observed experimentally (Fig. 4) could be partly attributed to a more positive MDP and decreased action potential amplitude (Supplementary Fig. 9A) rather than simply an increased repolarization rate, as might be interpreted from normalized optical recordings (Supplementary Fig. 9B).

Modeling showed that increased *G*_MFB-CM_ caused CMs to depolarize and MFB and CM potentials to become more similar, while increased light intensity made the MDP of both more positive (Fig. 6D). At the estimated *G*_MFB-CM_ and 500 ms CL pacing, MFB potential changed from − 23 mV for unconnected MFBs to − 67 mV (black dot on green curve, Fig. 6D), while depolarizing CMs from − 73 to − 71 mV (black dot on red curve, Fig. 6D). Application of light produced additional inward ChR2 current that depolarized MFBs to − 60 and − 53 mV and further depolarized CMs to –67 or –61 mV for I_0_ or 3*I_0_, respectively (blue dots at I_0_ and 3*I_0_, Fig. 6D). Higher light level (10*I_0_) produced more depolarized MDPs (− 42 and − 55 mV for MFBs and CMs, respectively; rightmost blue dots, Fig. 6D–E) as well as spontaneous beating (dashed lines, Fig. 6D–E). The inward sodium current in CMs, *I*_Na_, decreased from 199 pA/pF for CM-only cables to 154 pA/pF (black dot on purple curve, Fig. 6E) for ChR2-MFB/CM cables, and to 81 and 9 pA/pF with the further addition of I_0_ or 3*I_0_ light, respectively (blue dots on purple curve, Fig. 6E). The reduced inward current resulted in lower CV (black to blue dots, Fig. 6F). Sodium current was highly inactivated at higher light levels, resulting in calcium-mediated conduction during spontaneous beating, since calcium current, *I*_CaL_ was much less affected by changes in MDP (black to blue dots on gold curve, Fig. 6E inset). At 3*I_0_, peak *I*_CaL_ was 68% of peak *I*_Na_, whereas at 10*I_0_, it was 490% peak *I*_Na_ (Fig. 6E inset).

**Figure 7:**
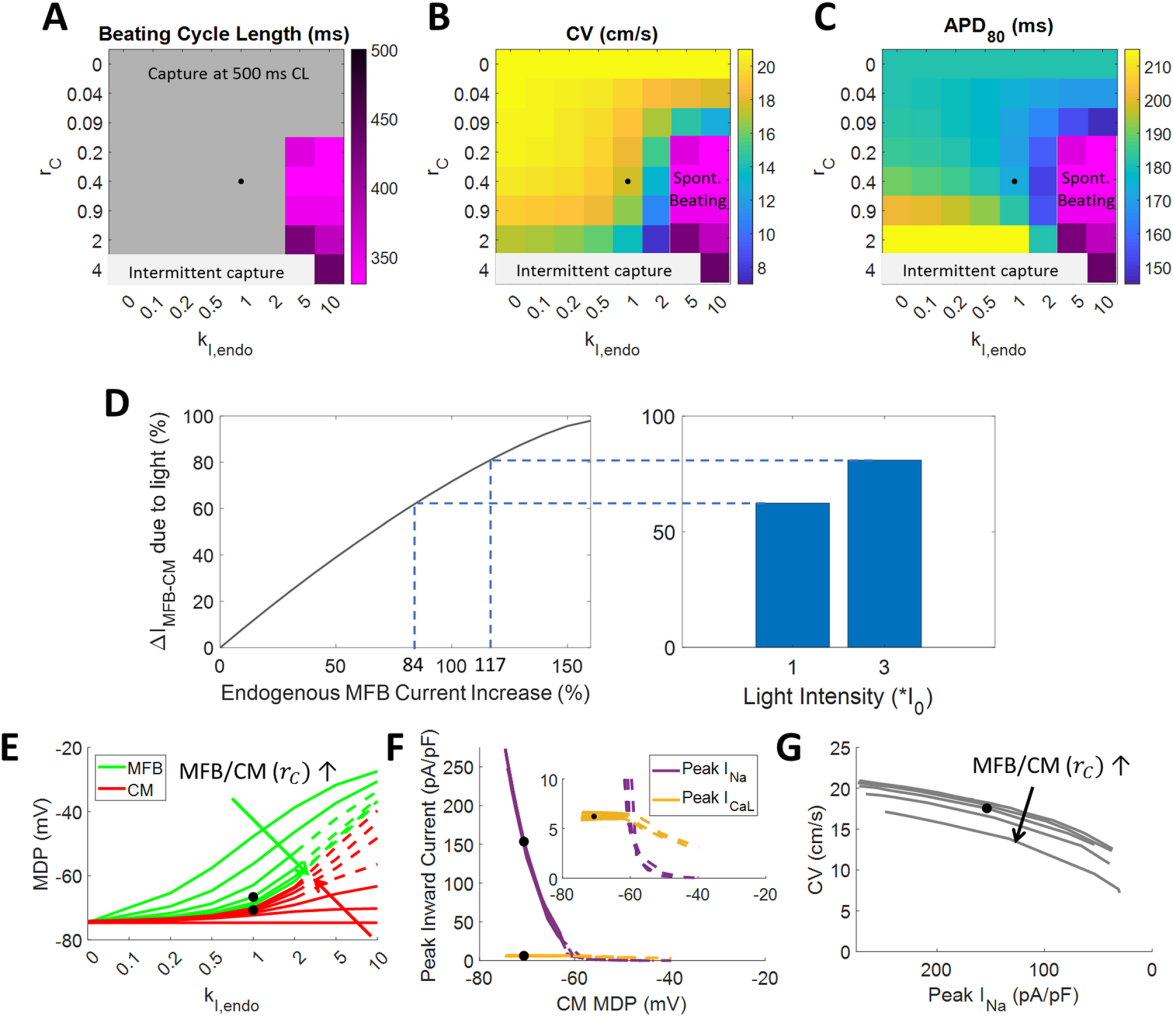
Modeling effects of changes in MFB/CM ratio and MFB currents. MFB-CM conductance was scaled with MFB capacitance ratio (*r*_C_), to model changes in MFB/CM ratio. Changes in endogenous MFB current scaling (*k*_I,endo_, per MFB capacitance) were also modeled. Color maps show beating cycle length (**A**), conduction velocity (**B**), and APD_80_ (**C**), at different MFB-CM conductances and light intensities. Parameters are varied in third-log_10_ increments around the baseline values. Gray region in A denotes that spontaneous beating, if present, was slower than the 500 ms paced CL. CV and APD_80_ were not calculated for the purple region in B and C since spontaneous beating prevented capture at 500 ms CL pacing. Light gray region denotes that beating occurred at less than the 500 ms paced cycle length due to intermittent capture, sometimes with spontaneous beating. Black dots indicate modeled values without light at the experimentally plated cell ratio of 0.4 and at baseline MFB conductance. (**D**) Modeling the effects of ChR2 current versus increased endogenous MFB currents showed that the light intensities used in experiments (I_0_ and 3*I_0_) resulted in the same MFB-CM current during diastole as an 84% and 117% increase in endogenous MFB currents, respectively. (**E**) Maximum diastolic potential of MFBs (green) and CMs (red). MFB line at MFB/CM capacitance ratio = 0 not shown since this implies the absence of MFBs. (**F**) Peak inward sodium current (purple) and L-type calcium current (gold) versus CM maximum diastolic potential. Inset shows same data with an expanded scale. (**G**) Conduction velocity versus peak sodium current. (**E–G**) MFB-CM capacitance ratio starts at 0 then increases by third-log_10_ increments from 0.04 to 2. Solid lines show conditions that allowed capture at the 500 ms paced CL. Dashed lines show conditions that caused spontaneous beating in excess of the pacing rate. Black dots indicate modeled values without light at the experimental MFB/CM cell ratio of 0.4 and at baseline MFB current level (i.e. the same as the black dots in Fig. 6).

To estimate effects of changes in MFB/CM ratio, we examined the effects of changing the MFB/CM capacitance ratio, *r*_C_, in tandem with *G*_MFB-CM_, since both are expected to directly vary with MFB/CM ratio. We also modeled the effects of different levels of endogenous MFB currents, by scaling them by a factor of *k*_I,endo_ (Supplementary Tables 2–3), where *k*_I,endo_ = 1 represents their normal, baseline values. In doing so, we found that increasing *r*_C_ reduced excitability, resulting in intermittent capture at *r*_C_ = 4 (Fig. 7A). In our model, there was no spontaneous beating faster than the 500 ms paced rate at *k*_I,endo_ = 1, regardless of *r*_C_; however, it did occur at *k*_I,endo_ > 2 (Fig. 7A and Supplementary Fig. 6B). Furthermore, while a minimum *r*_C_ was necessary to produce spontaneous beating, further increase of *r*_C_ generally caused progressive slowing of spontaneous beating (Fig. 7A). Increasing either *r*_C_ or *k*_I,endo_ could slow conduction significantly (to < 8 cm/s from 21 cm/s, Fig. 7B). Also, increasing *r*_C_ could either increase or decrease APD_80_, while increased *k*_I,endo_ always decreased it (Fig. 7C). Comparing the effects of ChR2 current and increased endogenous MFB currents (*k*_I,endo_ > 1), the model showed that the light intensities used in experiments (I_0_ and 3*I_0_) resulted in MFB-CM current during diastole equal to that produced by an 84% and 117% increase in endogenous MFB current, respectively (Fig. 7D).

Modeling also showed that as with *G*_MFB-CM_, increased *r*_C_ caused MFB and CM potentials to become more similar, while increased MFB currents made the overall MDP of both more positive (Fig. 7E). Plots of peak *I*_Na_ and *I*_CaL_ versus CM MDP almost completely overlap at different *G*_MFB-CM_ (Fig. 6E) and *r*_C_ (Fig. 7F), showing that these currents are essentially determined only by MDP. However, plots of CV versus peak I_Na_ show that these curves do not overlap and that CV decreases with increased *G*_MFB-CM_ (Fig. 6F) or *r*_C_ (Fig. 7G) independently of peak I_Na_. Thus, CV is decreased both by reduced *I*_Na_ caused by elevated resting potential as well as by the loading effects of MFBs, including capacitance (illustrated clearly in the first column of Fig. 7B, where MFB currents = 0).

## Discussion

Since the initial findings of Miragoli et al.^18^ showing that addition of MFBs to CM cultures causes MDP elevation, conduction slowing, and spontaneous beating, a number of studies have attributed MFB-induced conduction slowing to electrical coupling between CMs and MFBs^6,7^. Interventional experiments have knocked down Cx43 in MFBs, and showed that doing so increased CV in co-cultures, compared with CMs co-cultured with unaltered MFBs, to provide direct evidence of an electrical mechanism for CV reduction by MFBs^11,22^. However, decreasing Cx43 expression also inhibits fibroblast differentiation to MFBs^23,24^ and presumably the number of MFBs present, so that CV would still increase even if MFB-induced suppression of CV is by non-electrical means (e.g., paracrine or mechanical signaling). Studies that dynamically and specifically alter the electrophysiology of MFBs and monitor the subsequent changes in CM electrophysiology can circumvent such confounding effects. Previous studies have shown that CM electrophysiology can be modulated by acutely altering exogenous potassium current in co-cultured 3T3 fibroblasts^25,26^. Another used 3T3 fibroblasts transduced with ChR2 and applied mW/mm^2^ light flashes to pace CMs^27^. One other study used sphingosine-1-phosphate to increase MFB inward current, and found that it suppressed CM excitability in co-cultures with MFBs, but not CMs alone^28^.

In this study, light was used to produce steady inward current specifically in ChR2-transduced cardiac MFBs. MFBs were sufficiently connected electrically to CMs for their coupling to produce diastolic depolarization (Fig. 2A and 4A), spontaneous focal beating (Fig. 2 and Supplementary Fig. 2), conduction slowing (Fig. 3), decreased APD_80_ (Fig. 4A–B), and decreased upstroke rate (Fig. 4C), all of which can contribute to arrhythmia in the context of fibrosis. While ChR2 is not naturally present in MFBs, it is similar to the TRP channels that are upregulated during MFB differentiation^29^ in that it is a relatively non-selective cation channel^15^, and our modeling shows that at the light levels used experimentally, the current it produces has a magnitude similar to that of endogenous MFB channels (Fig. 7D). The rapid time scale of these changes (within seconds, the time interval between measurements) eliminates the possibility that changes in cardiac ion channel expression in response to the presence of MFBs underlie these effects. Additionally, the absence of changes in force generation with application of light to ChR2-MFBs (Supplementary Fig. 3) rules out the possibility that CV slowing occurred secondary to acute changes in MFB tugging forces^30^. The fact that light had no effect on control MFB co-cultures (Figs. 2–4) also supports the notion that CV effects were due to light-induced ChR2 current and not to off-target effects such as heating or photochemical reactions. While confocal imaging suggests that the observed electrical connection between MFBs and CMs is due to Cx43 (Fig. 1F–H), other possibilities, including tunneling nanotubes^31^, remain. Furthermore, in addition to electrical mechanisms, paracrine and mechanical signaling may also contribute to MFB effects, especially changes in APD_80_, which we found decreased significantly in the presence of ChR2-MFBs in our experiments (from 191 ± 22, n = 14 to 165 ± 28 ms, n = 8; p = 0.03), but not in our model (from 181 to 173 ms, Fig. 6C).

The effects of addition of MFBs to CM syncytia have been previously explored experimentally^6,7^ and computationally^32^. Although experimental data have been derived mostly from cultured neonatal rat ventricular CMs, computational studies have generally relied on CM models of other species. In this work, ChR2 current^33^ was added to a MFB model^34^ parameterized directly from data from cultured neonatal rat MFBs^13^, which was coupled to a modified neonatal rat ventricular CM model^17^ (Supplementary Tables 2–7) to enable *G*_MFB-CM_ to be estimated. This approach extends the OptoGap method previously adopted by the authors^35^. Examination of different values of *G*_MFB-CM_ and light intensities showed that, provided *G*_MFB-CM_ is large enough, all of the phenomena seen in our experiments occur, including spontaneous beating (Fig. 6A), conduction slowing (Fig. 6B), and APD_80_ reduction (Fig. 6C). We then determined the syncytial *G*_MFB-CM_ that best matched our experimental data. While previous studies have used FRAP to determine the relative coupling between CMs and other cells, including MFBs^36^, these cannot be directly translated to a value of electrical conductance^35^. Dual-cell patch clamp has been used to quantify the electrical connection between CM and MFB cell pairs^13,14^, but estimated values have varied over a very wide range (0.31^14^ to 165 nS^13^). Also, cells grown in isolation have structural and electrophysiological properties that differ from cells grown in a syncytium^37^. Furthermore, dual-cell patch clamp measures the conductance between adjacent cells spread on a substrate, where because of the small height of the cells relative to their area, only a small fraction of the cell surface is available for connection, which is not the case for cells in 3-D tissue. Indeed, this limited interface also occurs in co-cultures of cells intermixed in 2-D. In this study MFBs were layered on top of CM monolayers, so that the MFB-CM interface was effectively in 3-D and occurred over a large area, allowing us to estimate a syncytial *G*_MFB-CM_ of 5.3 nS/CM in a geometry more similar to that in vivo, and resulting in excellent agreement between our model and experiments (Supplementary Figs. 7 and 8, Supplementary Table 8).

We also proceeded to model the effects of important changes that can occur in fibrosis – increased *G*_MFB-CM_, increased *r*_C_ due to increased number of (myo)fibroblasts, and increased *k*_I,endo_. The results showed how these contribute to arrhythmogenic spontaneous beating (Fig. 6A and 7A) and conduction slowing (Fig. 6B and 7B). The model also substantiates the previously proposed mechanism^38^, as well as our experimental findings, that endogenous (Fig. 7E) or exogenous (Fig. 6D) currents in MFBs can depolarize CMs, thereby inactivating their sodium channels (Fig. 6E and 7F), reducing upstroke rate (Fig. 4C), and slowing conduction (Fig. 6F and Fig. 7G). It also shows that increased MFB inward current causes spontaneous beating (Fig. 6A and 7A), which has the highest rate at *r*_C_ = 0.2–0.9. This intermediate range of values suggests that moderate levels of fibrosis produce the greatest risk of ectopic beating by this mechanism. During spontaneous beating, the model shows conduction is mediated significantly by calcium current (Fig. 6E, inset) due to block of sodium current, in agreement with a previous study that found little effect of the sodium channel blocker TTX on CV in CM strands co-cultured with large numbers of MFBs^18^.

Our findings show that the CM MDP and the relative amount of MFB to CM currents are important determinants in effects of MFBs on CV and spontaneous beating. Our neonatal rat CM model has a more positive MDP and lower resting membrane resistance than in adult rat or human cells, which makes them more susceptible to MFB electrical effects. However, elevated MDP and reduced *I*_K1_ current (reducing resting CM current) have been reported in heart failure^39^, which is often a comorbidity of fibrosis^4,5^, suggesting that our model may well apply in this case.

Our findings complement two recently published studies which used 3T3 fibroblasts transduced with a depolarizing or hyperpolarizing opsin to investigate the relative contributions of current sinking vs. diastolic depolarization effects of the fibroblasts on neonatal rat CM electrophysiology^40^, and show that light-activated fibroblasts can increase or decrease beating rate in human pluripotent stem cell-derived CMs, especially after fibroblast differentiation to MFBs by TGF-β1^41^. Our work used isolated neonatal rat cardiac myofibroblasts, which have different electrophysiology than 3T3 fibroblasts^42^, together with the depolarizing opsin ChR2 to produce spontaneous beating, conduction slowing, decreased upstroke velocity, and reduced APD_80_, and estimate *G*_MFB-CM_ in a syncytium across a 3-D interface between MFBs and CMs. Previous studies^43,44^ have also probed electrical connections between MFBs and CMs in vivo by creating genetically engineered mice that expressed an optogenetic voltage sensor specifically in non-CMs, and found time-varying signals specifically near sites of injury, suggesting that non-CMs can electrically connect to CMs in those areas. However, this does not necessarily demonstrate the *G*_MFB-CM_ is large enough to significantly affect CM electrophysiology, since non-CMs are better voltage followers than drivers when coupled to CMs because of their higher sarcolemmal resistance and lower sarcolemmal currents^35^. Indeed, our modeling suggests that MFBs have significant action potential-like deflections in membrane voltage even under conditions where they have little effect on CM electrophysiology. For example, when light intensity = 0 and *G*_MFB-CM_ = 0.53 nS/CM (one-tenth that suggested by this study), MFB amplitude is 55% of CM amplitude (Supplementary Fig. 6A), while CV decreases by only 7% (Fig. 6B) and APD_80_ by only 3% (Fig. 6C). Therefore, using a similar design with an optogenetic actuator, as done in this study, instead of an optogenetic sensor may be better suited to determine whether *G*_MFB-CM_ is large enough to cause arrhythmogenic conduction slowing and spontaneous beating in vivo.

## Conclusion

This study used optogenetic actuation of inward current in myofibroblasts to show that they can acutely cause spontaneous beating, conduction slowing, decreased upstroke rate, and decreased action potential duration in co-cultured cardiomyocytes, clearly demonstrating functional electrical coupling between these cells. Computational modeling of the experiments allowed the estimation of myofibroblast-myocyte coupling in a syncytium in which myofibroblasts and cardiomyocytes interact in 3-D. It also demonstrated how increased strength of myofibroblast electrical coupling to cardiomyocytes, increased myofibroblast/myocyte capacitance ratio, and increased myofibroblast current levels, all of which may occur in fibrosis, can work in tandem to generate a proarrhythmic reduction in conduction velocity and increase in spontaneous beating.

## Methods

An expanded description of the methods is available in the supplementary material.

### Cell culture

This study is in compliance with the ARRIVE guidelines for in vivo study on animals. All animal procedures were approved by the Johns Hopkins Animal Care and Use Committee and were performed in compliance with guidelines of federal and state laws and regulations. Neonatal rat ventricular CMs were produced as described previously^45^, with minor modifications, and plated onto coverslips coated with 25 μg/mL fibronectin at 1 million cells per well for a 12-well plate or 500,000 per well for a 24-well plate (approximately 250,000/cm^2^). During isolation, CMs were separated from fibroblasts using two 1-h preplating steps. Fibroblasts from the first preplate were passaged twice. Some were transduced during the second passage with ChR2-YFP adenovirus at a multiplicity of infection of 2,000, as determined by experiments (Supplementary Fig. 10), with media changed 4–6 h later to remove virus as described previously^46^. Transduced and non-transduced fibroblasts were treated with 5 ng/mL TGF-β1 (R + D Systems) for 2–3 days to differentiate them into MFBs.

### Co-culture, imaging, and optical mapping

ChR2-MFBs or non-transduced MFBs were added to 4 to 5-day-old CM monolayers at 400,000/well in 12-well plates or 200,000/well in 24-well plates (approximately 100,000/cm^2^), giving a MFB:CM cell ratio of 0.4. High levels of TGF-β1 treatment were used to irreversibly differentiate fibroblasts into MFBs^47^ to ensure maintenance of an MFB phenotype without application of exogenous TGF-β1 during co-culture, which could have directly affected CMs. On days 5–8, co-cultures were imaged under phase contrast and fluorescence microscopy (Eclipse TE2000U, Nikon) to examine their morphology and continued expression of ChR2, and then placed in a custom optical mapping system^48^ and stained for 5 min with 35 μM of the voltage-sensitive dye di-4-ANBDQBS (obtained from Dr. Leslie Loew, University of Connecticut), which is excited by red light (λ = 655 nm)^49^ and therefore spectrally different than excitation of ChR2^15,50,51^. Tyrode’s solution at 35 °C was then continuously flowed over the cells. Pacing threshold was determined to within 1 V, and cells were point paced at 1.1 × threshold for 5 min at 500 ms CL to reach steady state. A baseline optical recording was taken, and then continuous blue light (λ = 448 nm) was applied across the entire monolayer to activate ChR2 channels for approximately 2 s before and throughout the duration of a 2 s recording, after which the light was switched off, and a post-activation recording was collected within seconds. This was done for different light intensities, starting at approximately the lowest intensity for which changes could be detected (I_0_ = 0.0057 mW/mm^2^), and increasing to 3*I_0_ and 10*I_0_, at which point 6/7 samples beat spontaneously faster than the paced rate. Co-cultures were fixed and stained for α-actinin (Sigma), α-SMA (DAKO), connexin43 (Cx43) (Sigma), YFP (Invitrogen GFP Ab), and/or DAPI before confocal imaging (LSM 710NLO-Meta, Zeiss).

### Mathematical model

Experimental data were modeled in MATLAB (The MathWorks) using a modified version of the Korhonen model of neonatal rat ventricular CMs^17^ (see Fig. 5, expanded methods, Supplementary Fig. 11 and Supplementary Tables 2–7 for model details). With the modifications, the model had a much better fit to the experimental data (Supplementary Figs. 4, 7 and 8 and Supplementary Table 8). CMs were connected via a lumped gap junction to a lumped MFB unit, with currents defined by the Sachse fibroblast model^34^ with current conductances fitted to the I/V curve measured by Salvarani et al. for TGF-β1-treated MFBs^13^ (Fig. 5A). In addition to endogenous currents, the Williams model for ChR2 current^33^ was added to MFBs. MFBs were considered to have similar capacitance per cell as CMs, as done previously^52^, and so *r*_C_ = 0.36, similar to the cell number ratio in experiments. Each CM was 50 μm long, and had a 50 µm-long interface with the following cell, based on previous measurements^52^. A 30-cell-long 1-D cable of ChR2-MFB/CM cell pairs was modeled (Fig. 5B and Supplementary Tables 2–4). Based on previous data which found conductance between two CMs to be 7.7 nS/μm^13^, neighboring CMs were connected by lumped gap junctions with a conductance of 50 μm*7.74 nS/μm = 387 nS.

### Data processing and statistics

Optical mapping data were processed by custom MATLAB software. Co-cultures with initial CV below 10 cm/s or that could not be paced at 500 ms CL (either due to inexcitabality or spontaneous beating faster than the paced CL) were excluded from analysis. Only samples that were not beating faster than the paced 500 ms CL were included in CV and APD analysis. Confocal images were processed by FIJI^53^ and Zen Black (Zeiss) software. Background values from empty wells were subtracted from plate reader measurements. All data are presented as mean ± SD. Paired or unpaired t-tests with unequal variances were used to determine statistical differences, where appropriate. Differences were considered statistically significant at p < 0.05. Additional statistics on experimental data are shown in Supplementary Table 1.

## Supplementary Information

Supplementary Information

## Data Availability

The datasets generated and analyzed during the current study are available from the corresponding author on reasonable request.
