# Supplementary material for: Optogenetic current in myofibroblasts acutely alters electrophysiology and conduction of co-cultured cardiomyocytes
Source: Sci Rep. 2021 Feb 24;11:4430. doi: 10.1038/s41598-021-83398-4 (PMC7904933; doi:10.1038/s41598-021-83398-4)
Supplement: Supplementary file 1 — Supplementary Information [file 41598_2021_83398_MOESM1_ESM.docx]

**Optogenetic current in myofibroblasts acutely alters electrophysiology and conduction of co-cultured cardiomyocytes**

Geran M. Kostecki^1^, Yu Shi^2^, Christopher S. Chen^3,4^, Daniel H. Reich^2^, Emilia Entcheva^5^, Leslie Tung^1^*

^1^Department of Biomedical Engineering, Johns Hopkins University, Baltimore, MD, USA

^2^Department of Physics and Astronomy, Johns Hopkins University, Baltimore, MD 21218, USA

^3^Biological Design Center, Department of Biomedical Engineering, Boston University, Boston, MA

^4^Wyss Institute for Biologically Inspired Engineering, Harvard University, Boston, MA

^5^Department of Biomedical Engineering, George Washington University, Washington, DC USA

*Corresponding Author:

Leslie Tung, Ph.D.

Department of Biomedical Engineering

720 Rutland Ave.

Rm. 703, Traylor Bldg.

Baltimore, MD 21205

office: 410-955-7453

email: ltung@jhu.edu

**Expanded Methods**

*Cell culture*

This study is in compliance with the ARRIVE guidelines for *in vivo* study on animals. All animal procedures were approved by the Johns Hopkins Animal Care and Use Committee and were performed in compliance with guidelines of federal and state laws and regulations. Cell culture media consisted of Medium 199 (Gibco) supplemented with 1% HEPES buffer solution (Gibco), 1% MEM non-essential amino acids (Gibco), 20 µM glucose (Millipore-Sigma), 2 mM L-glutamine (Life Technologies), 4 µg/mL vitamin B-12 (Millipore-Sigma), 1% penicillin (Millipore-Sigma) and 10% fetal bovine serum (FBS, Millipore-Sigma). The hearts of a litter of 2-day-old Sprague-Dawley rats (Harlan) were excised, cut into 4-6 pieces, rinsed twice in HBSS, placed in 40 mL 0.25 mg/mL filtered trypsin (Millipore-Sigma) in HBSS solution in a sealed glass container, and rotated at 110 rpm at 4°C overnight (13-16 h). The next day as much trypsin solution as possible (without removing cells) was pipetted out, and the remainder quenched by adding 25 mL 10% FBS media. The tissue was agitated in a warm water bath for 3 min using an orbital shaker at 150 rpm. Then, excess media was removed, and 9 mL of 1 mg/mL collagenase (Worthington) in HBSS was added before sealing the glass container holding the tissue and placing it back in the warm water bath for 3 min. After this, excess solution was removed and discarded. Another 9 mL of collagenase solution was added, the glass container was sealed and rotated in the warm water bath again for 3 min, the cells were pipetted up and down three times, and the excess solution was removed and placed in a 15 mL centrifuge tube with 4 mL cold HBSS, which was placed in ice to quench collagenase activity. This was done 3 more times. The tubes were centrifuged at 3,000 rpm for 8 min, excess solution was aspirated, and cells were pipetted out into 10 mL cold HBSS. They were then triturated by pipetting before being passed through a 40 µm nylon cell strainer (BD Falcon). Cells were then centrifuged again for 5 min at 1,000 rpm and 1 min at 3,000 rpm, before aspirating out the HBSS, resuspending in 15 mL 10% FBS media, and preplating for one hour in a 175 cm^2^ tissue culture flask (Sarstedt) to isolate fibroblasts. They were then preplated again in 25 mL 10% FBS media in a 175 cm^2^ flask (Sarstedt) for one hour. The supernatant, consisting of purified cardiomyocytes (CMs), was removed, and cells were counted using a hemocytometer (Thermo Fisher Scientific). CMs were plated onto coverslips coated with 25 μg/mL fibronectin at 1 million per well for a 12-well plate or 500,000 per well for a 24-well plate (approximately 250,000/cm^2^).

Myofibroblasts (MFBs) used in experiments were produced by passaging cells from the first preplate twice (on day 4-5 and 11-12) and transducing some of them with ChR2-YFP adenovirus^1^ as described previously^2^ at a multiplicity of infection (MOI) of 2,000 during the second passage to produce ChR2-MFBs, with media changed 4-6 hours later to remove virus. Cells were treated with 5 ng/mL TGF-β1 (R+D Systems) four days after transduction to fully differentiate them into MFBs. These procedures were also followed for initial pilot experiments except that fibroblasts from the preplate were plated onto 0.1% gelatin-coated 24-well #0 glass-bottom plates (Cellvis) at 200,000/well, and 0, 1,000, 2,000, or 5,000 MOI of virus was added to some of the wells. Eight days after plating fibroblasts were fixed and stained for YFP (Invitrogen GFP Ab) and DAPI using standard protocols. Cells were then imaged using a confocal microscope (LSM 710NLO-Meta, Zeiss). These pilot studies showed >50% of cells were transduced at all tested MOI (Supplementary Fig. 10A). Fluorescence measurements using a plate reader after fixing and staining for YFP showed a statistically significant increase in fluorescence between 2,000 and 1,000 MOI, and only a small, non-significant further increase with 5,000 MOI (Supplementary Fig. 10B), possibly due to cell death. Hence, we chose to use 2,000 MOI for the experiments presented in this study.

*Co-culture, imaging, and optical mapping*

ChR2-transduced MFBs (ChR2-MFBs) or untransduced MFBs were added to 4 to 5-day-old CM monolayers at 400,000/well in 12-well plates or 200,000/well in 24-well plates (approximately 100,000/cm^2^), giving a MFB:CM cell ratio of 0.4. To limit direct TGF-β1 effects on CMs, TGF-β1 was not present during co-culture. On days 5-8, co-cultures were imaged under phase contrast and fluorescence microscopy (Eclipse TE2000U, Nikon) to examine their morphology and continued expression of ChR2, then placed in a custom optical mapping system^3^, modified to optically stimulate ChR2 as well as record transmembrane voltage using the voltage-sensitive dye di-4-ANBDQBS (from Dr. Leslie Loew, University of Connecticut) which is excited by red light^4^ (λ=655 nm) and therefore can be excited without significantly opening ChR2 channels^5,6^. To do this, red (λ=655 nm) and blue (λ=448 nm) LED modules (Luxeon SinkPAD-II Rebel 7 LED Round Modules) were arranged perpendicularly, with a dichroic mirror (λ= 475) between them at a 45° angle. The emission filter at the bottom of the mapping chamber consisted of two stacked 717 nm Wratten filters (Kodak, 717FWP7575) affixed to the bottom of a 1 mm-thick glass slide using clear tape. Cells were stained for 5 minutes with 35 μM of di-4-ANBDQBS. Tyrode’s solution (1.8 mM CaCl2, 5 mM glucose, 5 mM HEPES, 1 mM MgCl2, 5.4 mM KCl, 135 mM NaCl, and 0.33 mM NaH2PO4 in ddH2O pHed to 7.37 with NaOH, all reagents from Sigma-Aldrich) at 35°C was then continuously flowed over the cells. The pacing threshold with 10 ms pulse width was determined to within 1 V, and cells were point paced at 1.1x threshold for 5 min at 500 ms to reach steady-state. A baseline optical recording was taken, then continuous blue light was applied across the entire monolayer to activate ChR2 channels for approximately two seconds before and throughout the duration of a two second recording, after which the light was switched off, and a post-ChR2 activation recording was collected within seconds. This was done for different light intensities, starting at approximately the lowest intensities for which changes could be detected (I_0_=0.0057 mW/mm^2^), and increasing to 3*I_0_ and 10*I_0_, at which point most samples beat spontaneously faster than the paced rate.

*Immunostaining*

After optical mapping, co-cultures were fixed in 4% paraformaldehyde, permeabilized by applying 0.2% Triton X-100 (Sigma-Aldrich) for 5 min and blocked using 10% goat serum (Life Technologies) in PBS for 25 min at room temperature. They were then incubated in primary antibodies against α-actinin (Sigma), α-smooth muscle actin (DAKO), connexin43 (Sigma), YFP (Invitrogen GFP Ab), and/or DAPI diluted in Antibody Diluent (Dako) overnight at 4°C. They were then washed three times for 5 min with TBS-T, then incubated with a 1:200 dilution of Alexa Fluor-conjugated goat secondary antibodies (Invitrogen) and DAPI (30 µM in PBS) in antibody diluent for 1 hour at room temperature. Finally, samples were washed again 3 times with TBS-T and mounted on microscope slides using ProLong Gold Antifade (Invitrogen) for confocal imaging (LSM 710NLO-Meta, Zeiss).

*Myofibroblast size and strain energy measurements*

MFBs were produced as described above, except after the second passage, 10,000 cells were plated in a 35-mm dish onto force-sensing micropost arrays produced as described previously^7^. 20 h after seeding, MFBs were continuously imaged via bright field using an inverted microscope (Nikon TE-2000) 60 s before application of 30 s high intensity (1.2 mW/mm^2^) blue light, through 60 s after it was turned off. Micropost images were analyzed by centroid fitting to track their positions $\vec{x}$^8,9^. The microposts’ undeflected positions $\vec{x}_{undeflected}$ were calculated by fitting to a hexagonal grid based on the measured positions of nearby microposts not in contact with a cell. The total bending strain energy of the microposts in contact with a cell,$E=\sum\frac{1}{2}k|\vec{x}-\vec{x}_{undeflected}|^{2}$, was used as a scalar metric to measure cellular static contractility.

*Mathematical model*

Experimental data was modeled in MATLAB (The MathWorks). Because only fibroblast and not MFB electrical models with time-dependent currents exist^10,11^, and their current-voltage curves differ dramatically from those measured in MFBs^12^, MFB currents were based on the Sachse time-dependent model for fibroblast currents^10^, with conductances adjusted to obtain a least-squares fit of the I/V curve measured by Salvarani, et. al.^12^ for MFBs differentiated by treatment with TGF-β1.(Fig. 5A). In addition to the endogenous currents, the Williams model for ChR2 current (I_ChR2_)^13^ was added to the MFB model (Supplementary Tables 2-4). MFBs were assumed to have the same capacitance as CMs on a cellular basis, as done previously^14^. Each CM was connected via a lumped gap junction to a MFB (Fig. 5B). Since the MFB:CM ratio was 0.4, we modeled one MFB connected to each CM, and simply multiplied its capacitance by 0.4. This was possible because on a scale on the order of tens of cells, the total membrane area of each cell type is the main determinant of CV, so a small number of large MFBs can be changed to a large number of small MFBs, as long as the total membrane area is maintained (see^14^, which found only ~5% decrease in CV across a wide range of MFB densities when decreasing MFB size by a factor of 4 but keeping total MFB membrane area constant). Each CM was 50 μm long, 50 µm wide, and 5 µm thick, based on previous measurements^14^, resulting in a CM capacitance of 60 pF, given $C_{m}$=.01 pF/µm^2^ (Fig. 5B and Supplementary Tables 2-3). Cell dimensions for calcium-handling equations were not changed from the original model, since the calcium handling is highly dependent on them, and the original model was calibrated based on those specific dimensions. A 30-cell-long (1.5 mm) 1-D cable of ChR2-MFB/CM cell pairs was modeled (Fig. 5B). Neighboring CMs were connected by lumped gap junctions with conductance $G_{CM-CM}=50 \mu m*7.74 \frac{nS}{\mu m}=387 nS$, based on measurements by Salvarani, et. al.^12^ (Fig. 5B and Supplementary Tables 2-3).

CM currents were modelled using a modified version of the Korhonen model for neonatal rat ventricular CMs^15^. Changes were made to make the model better match experimental data. L-type calcium channel calcium gates ($f_{Ca}$) were allowed to recover at voltages more depolarized than -60 mV (Supplementary Table 5). This prevents a step change in recovery rate which isn’t well justified physiologically. While this inability to recover at depolarized potentials has almost no effect on control cells during normal pacing, in the original model it almost completely prevents L-type calcium channel recovery when depolarized, preventing spontaneous beating. The I_Ks_ time constant was changed to the standard form $\tau_{nKs}=\frac{1}{\alpha_{n}+\beta_{n}}$ instead of being set to a constant, and the I_Ks_ α_n_ and β_n_ were changed so the maximum rates matched those found in guinea pig ventricle (Supplementary Fig. 11 and Supplementary Table 5), since this was the most similar species for which rates were available^16^. I_K1_ conductance was increased by a factor of 3.6; somewhat more than the factor of 2 increase used by Hou, et. al. based off of their experimental data^17^ (Supplementary Table 6). This was done to increase spontaneous beating, as well as decrease the resting potential (RP) to be in better agreement with other published data. Sodium and potassium concentrations were fixed at initial levels to speed simulations (Supplementary Table 5). Temperature was increased from 305 to 308 K to match experimental conditions (Supplementary Table 6). Finally, I_Na_ conductance was increased to 130 mS/µF to match the CV found in control samples (20.9±4.3 cm/s, n=14) (Supplementary Table 6). In the original model, it was scaled to generate a correct upstroke amplitude in a single cell, but this does not factor in the loading effect of downstream cells in the multicellular systems modelled in this work. Additional changes (Supplementary Tables 5-6) were made using optimization to minimize a scoring function (Supplementary Table 7) to match parameters from the original Korhonen model, as well as parameters measured experimentally in this study. Initially, optimization was performed in 2-cell cables to speed computation. After initial optimization, variables with little effect on the final score were reset to their original value, and the model was re-optimized using the remaining variables shown in Supplementary Tables 5 and 6, which sped computation and increased the likelihood of finding a global, rather than local, minimum. Finally, the full 30 cell system was run, optimizing only by varying *g_ChR2_*, *g_Na_*, and *G_MFB-CM_*, resulting in values of 2.9 nS/pF, 130 nS/pF, and 5.3 nS/CM. respectively (Supplementary Tables 3 and 6).

*Data processing and statistics*

Strain energy data were processed using Igor Pro (Wavemetrics), and optical mapping data were processed by custom MATLAB software. Optical traces were dedrifted, low pass filtered with a cutoff frequency of 32 Hz, and normalized. A 5-point derivative was calculated (1 ms time resolution), and activation time was calculated using the time of maximum upstroke. CV was calculated as the inverse of the maximal spatial derivative across a channel and its neighbors (1 mm channel resolution). Co-cultures with initial CV below 10 cm/s or that could not be paced at 500 ms CL (either due to inexcitabality or spontaneous beating faster than the paced CL) were excluded from analysis. Only samples that were not beating faster than the paced 500 ms CL were included in CV and APD analysis. In some cases, spontaneous beating at a rate faster than 500 ms CL began in the middle of experiments and further data could not be collected; in this case, the data already collected was still used. Overall, these criteria allowed the use of 19 of 39 samples (9/18 MFB co-cultures and 10/21 ChR2-MFB co-cultures) over four isolations. Treatment groups were not grouped together on culture plates and were plated in different wells for each isolation. Treatment groups were generally optically mapped in alternating order, with equal numbers from each group mapped each day in most cases. While the data collection was not blinded, a standard experimental protocol and exclusion criteria were followed, and data analysis was automated. No *a priori* sample size calculations were performed. Confocal images were processed by FIJI^18^ and Zen Black (Zeiss) software. Background values from empty wells were subtracted from plate reader measurements. All data are presented as mean±SD. Paired or unpaired t-tests with unequal variances were used to determine statistical differences, where appropriate. Differences were considered statistically significant at p<0.05. Additional statistics on experimental data are shown in Supplementary Table 8.

**References**

1. Klimas, A. *et al.* OptoDyCE as an automated system for high-throughput all-optical dynamic cardiac electrophysiology. *Nat. Commun.* **7**, 11542 (2016).

2. Yu, J. & Entcheva, E. Inscribing Optical Excitability to Non-Excitable Cardiac Cells: Viral Delivery of Optogenetic Tools in Primary Cardiac Fibroblasts. in *Optogenetics* **1408**, 303–317 (2016).

3. Lim, Z. Y., Maskara, B., Aguel, F., Emokpae, R. & Tung, L. Spiral wave attachment to millimeter-sized obstacles. *Circulation* **114**, 2113–21 (2006).

4. Matiukas, A. *et al.* Near-infrared voltage-sensitive fluorescent dyes optimized for optical mapping in blood-perfused myocardium. *Hear. Rhythm* **4**, 1441–1451 (2007).

5. Ambrosi, C. M., Klimas, A., Yu, J. & Entcheva, E. Cardiac applications of optogenetics. *Prog. Biophys. Mol. Biol.* **115**, 294–304 (2014).

6. Schneider, F., Grimm, C. & Hegemann, P. Biophysics of Channelrhodopsin. *Annu. Rev. Biophys.* **44**, 167–86 (2015).

7. Fu, J. *et al.* Mechanical regulation of cell function with geometrically modulated elastomeric substrates. *Nat. Methods* **7**, 733–736 (2011).

8. Crocker, J. C. & Grier, D. G. Methods of Digital Video Microscopy for Colloidal Studies Methods of Digital Video Microscopy for Colloidal Studies. *J. Colloid Interface Sci.* **179**, 298–310 (1996).

9. Shi, Y., Porter, C. L., Crocker, J. C. & Reich, D. H. Dissecting fat-tailed fluctuations in the cytoskeleton with active micropost arrays. *Proc. Natl. Acad. Sci.* (2019). doi:10.1073/pnas.1900963116

10. Sachse, F. B., Moreno, A. P. & Abildskov, J. A. Electrophysiological modeling of fibroblasts and their interaction with myocytes. *Ann. Biomed. Eng.* **36**, 41–56 (2008).

11. Andrew MacCannell, K. *et al.* A Mathematical Model of Electrotonic Interactions between Ventricular Myocytes and Fibroblasts. *Biophys. J.* **92**, 4121–4132 (2007).

12. Salvarani, N. *et al.* TGF-β1 (Transforming Growth Factor-β1) Plays a Pivotal Role in Cardiac Myofibroblast Arrhythmogenicity. *Circ. Arrhythmia Electrophysiol.* **10**, e004567 (2017).

13. Williams, J. C. *et al.* Computational Optogenetics: Empirically-Derived Voltage- and Light-Sensitive Channelrhodopsin-2 Model. *PLoS Comput. Biol.* **9**, 17–19 (2013).

14. Jousset, F., Maguy, A., Rohr, S. & Kucera, J. P. Myofibroblasts electrotonically coupled to cardiomyocytes alter conduction: Insights at the cellular level from a detailed in silico tissue structure model. *Front. Physiol.* **7**, 1–23 (2016).

15. Korhonen, T., Hänninen, S. L. & Tavi, P. Model of excitation-contraction coupling of rat neonatal ventricular myocytes. *Biophys. J.* **96**, 1189–1209 (2009).

16. Severi, S., Corsi, C., Rocchetti, M. & Zaza, A. Mechanisms of b -Adrenergic Modulation of I Ks in the Guinea-Pig Ventricle : Insights from Experimental and Model-Based Analysis. *Biophysj* **96**, 3862–3872 (2009).

17. Hou, L. *et al.* A major role for hERG in determining frequency of reentry in neonatal rat ventricular myocyte monolayer. *Circ. Res.* **107**, 1503–1511 (2010).

18. Schindelin, J. *et al.* Fiji : an open-source platform for biological-image analysis. *Nat. Methods* **9**, 676–682 (2012).

19. Miragoli, M., Salvarani, N. & Rohr, S. Myofibroblasts Induce Ectopic Activity in Cardiac Tissue. *Circ. Res.* 755–758 (2007). doi:10.1161/CIRCRESAHA.107.160549

20. Morrissette-McAlmon, J. *et al.* Adipose-derived perivascular mesenchymal stromal/stem cells promote functional vascular tissue engineering for cardiac regenerative purposes. *J. Tissue Eng. Regen. Med.* **12**, e962–e972 (2018).

21. McSpadden, L. C., Nguyen, H. & Bursac, N. Size and ionic currents of unexcitable cells coupled to cardiomyocytes distinctly modulate cardiac action potential shape and pacemaking activity in micropatterned cell pairs. *Circ. Arrhythmia Electrophysiol.* **5**, 821–830 (2012).

22. Miragoli, M., Gaudesius, G. & Rohr, S. Electrotonic modulation of cardiac impulse conduction by myofibroblasts. *Circ. Res.* **98**, 801–810 (2006).


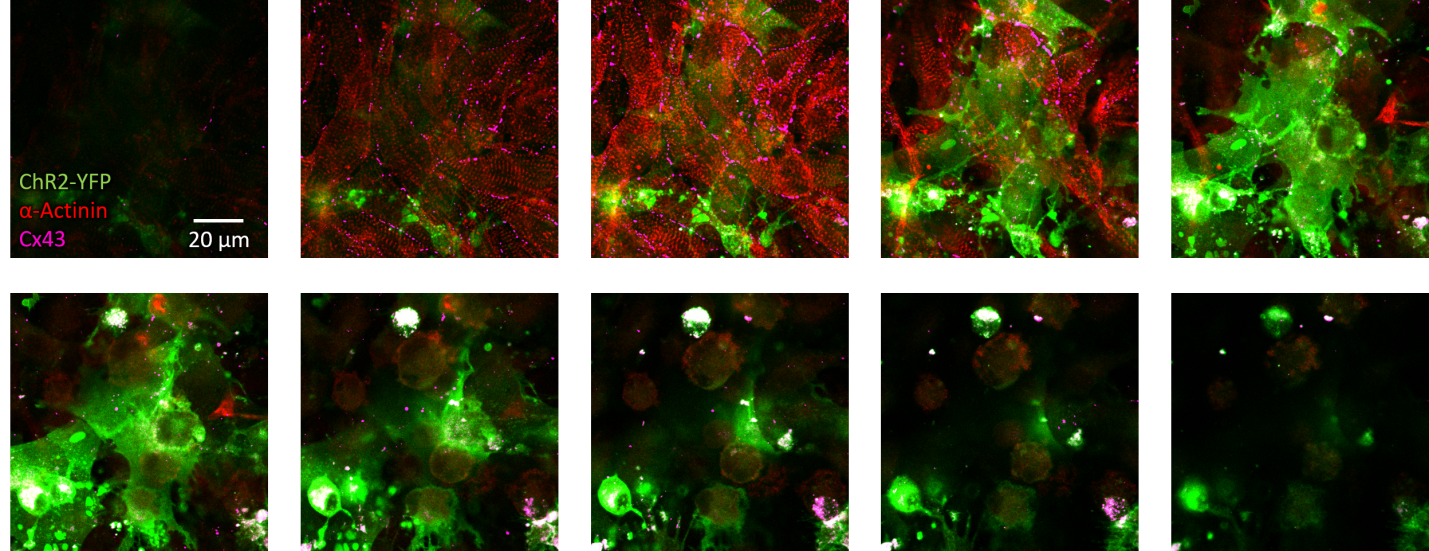


**Supplementary Figure 1.***Full z-stack as relates to Fig. 1F-H.*

ChR2-YFP (green) marks transduced MFBs, α-actinin (red) marks CMs, and violet shows connexin43 (Cx43). Image planes are 2 µm apart.


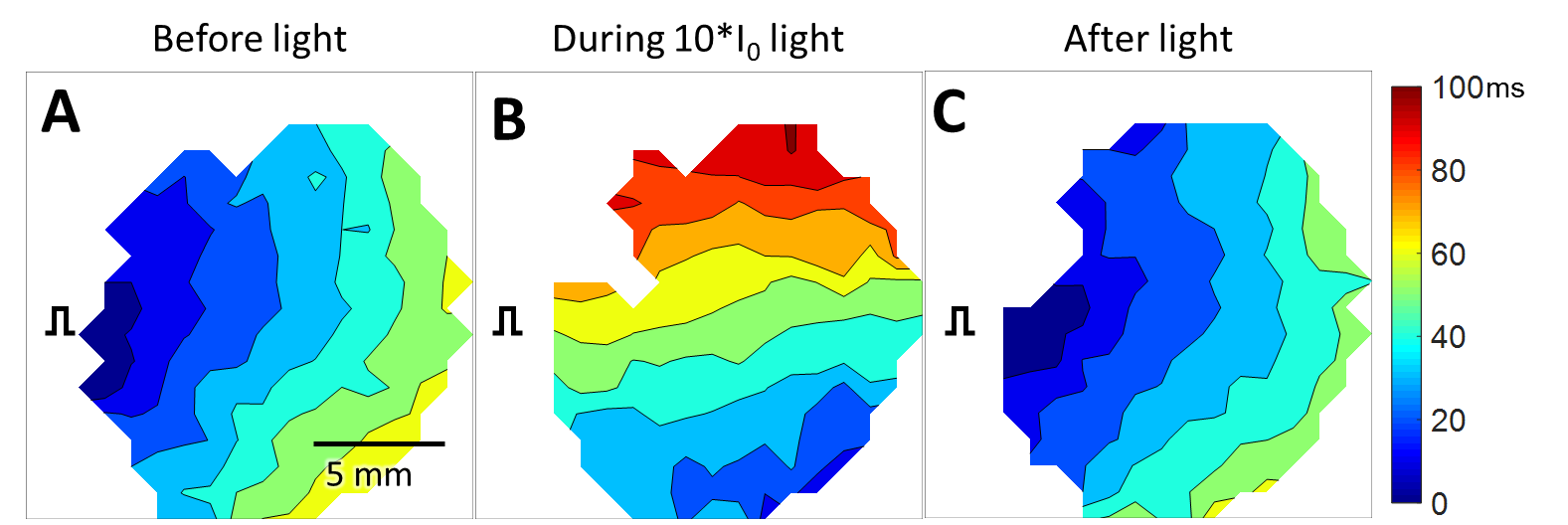


**Supplementary Figure 2.** *Activation maps of traces from Figure 2A.*

Activation maps of the ChR2-MF/CM co-culture shown in Figure 2A before (**A**), during (**B**), and after (**C**) application of 10*I_0_ blue light to produce inward current into ChR2-transduced MFBs. Isochrones are 10 ms apart; transition from blue to red is 100 ms. Pacing marker illustrates location of pacing.


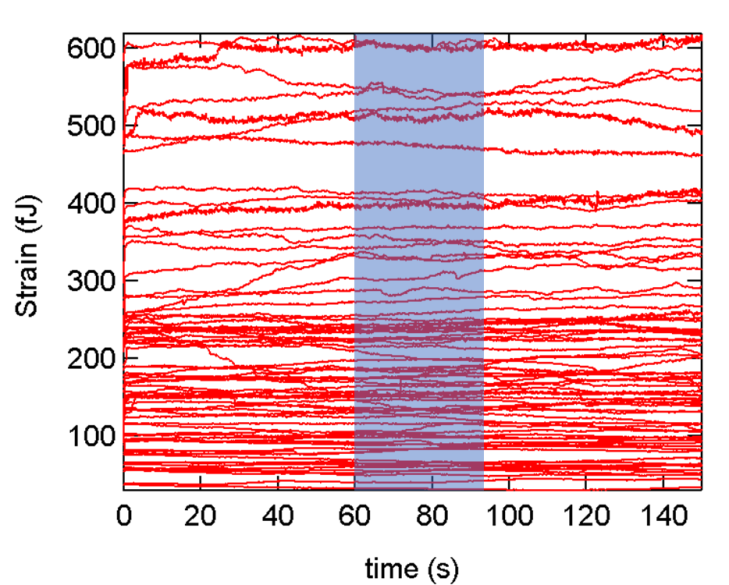


**Supplementary Figure 3.** *ChR2-transduced myofibroblast strain energy during excitation by light.*

Recordings of ChR2-MFB strain energy before, during (blue box), and after excitation with ChR2-saturating level of blue light (1.2 mW/mm^2^, equal to 210*I_0_). Each red line is the total strain energy for an individual MFB.


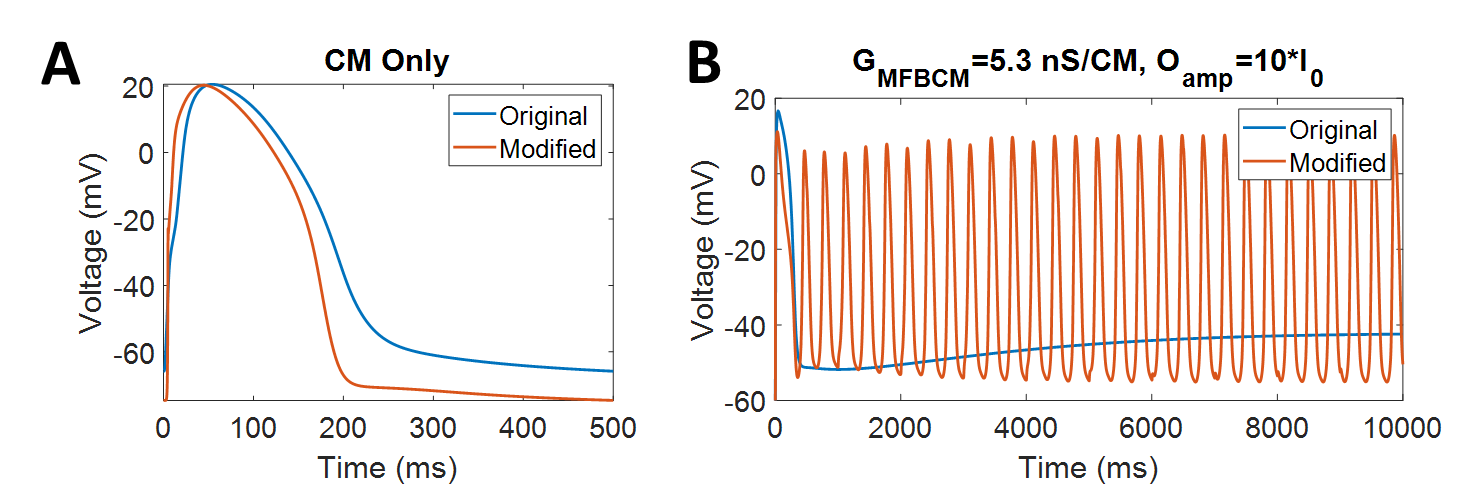


**Supplementary Figure 4.** *Modified model of cultured neonatal rat cardiomyocytes and ChR2-transduced myofibroblasts.*

**A.** Voltage traces from original (blue) and modified (orange) Korhonen model CMs in 30-cell cables paced at 500 ms CL. **B.** Voltage traces from original (blue) and modified (orange) CMs connected to ChR2-MFBs during light intensity of 10*I_0_.


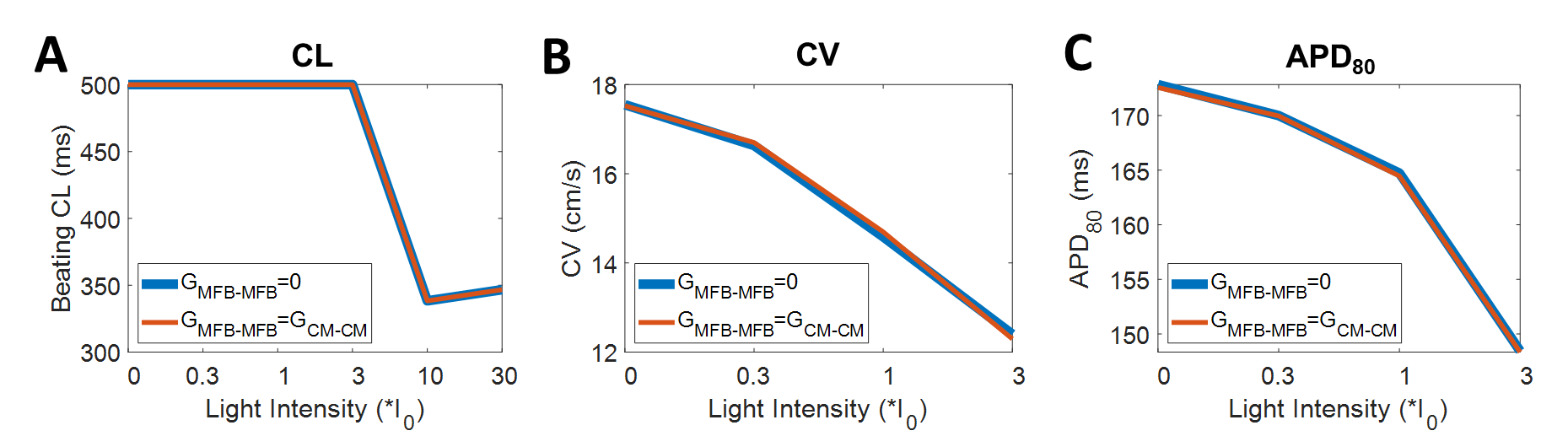


**Supplementary Figure 5.** *MFB-MFB coupling has little functional effect.*

While the model had *G_MFB-MFB_* = 0 (blue), choosing *G_MFB-MFB_* = *G_CM-CM_* (orange) had little effect on beating cycle length (**A**), conduction velocity (**B**), or APD_80_ (**C**).


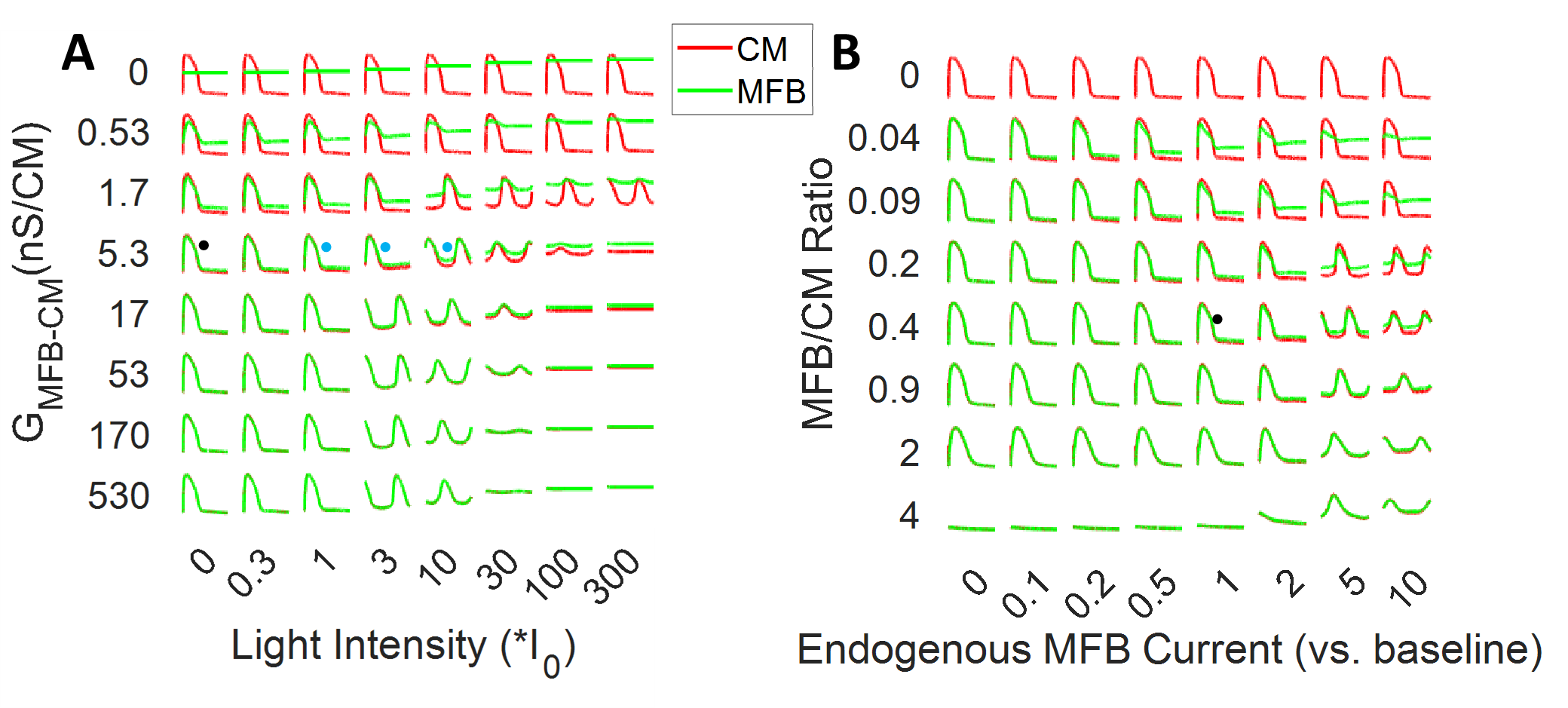


**Supplementary Figure 6.** *Myofibroblast and cardiomyocyte voltage traces from Figs. 6 and 7*

Traces are arranged in the same layout as the data in Fig. 6 (**A**) and Fig. 7 (**B**). Red shows CM traces, and green shows MFB traces. There are no green MFB traces in the first row of B since a MFB/CM ratio of 0 implies no MFBs are present. Black dot indicates modeled values without light, and blue dots indicate modelled values at light power levels used in experiments, all at the experimentally plated ratio of 0.4 and at the estimated MFB-CM conductance of 5.3 nS/CM.


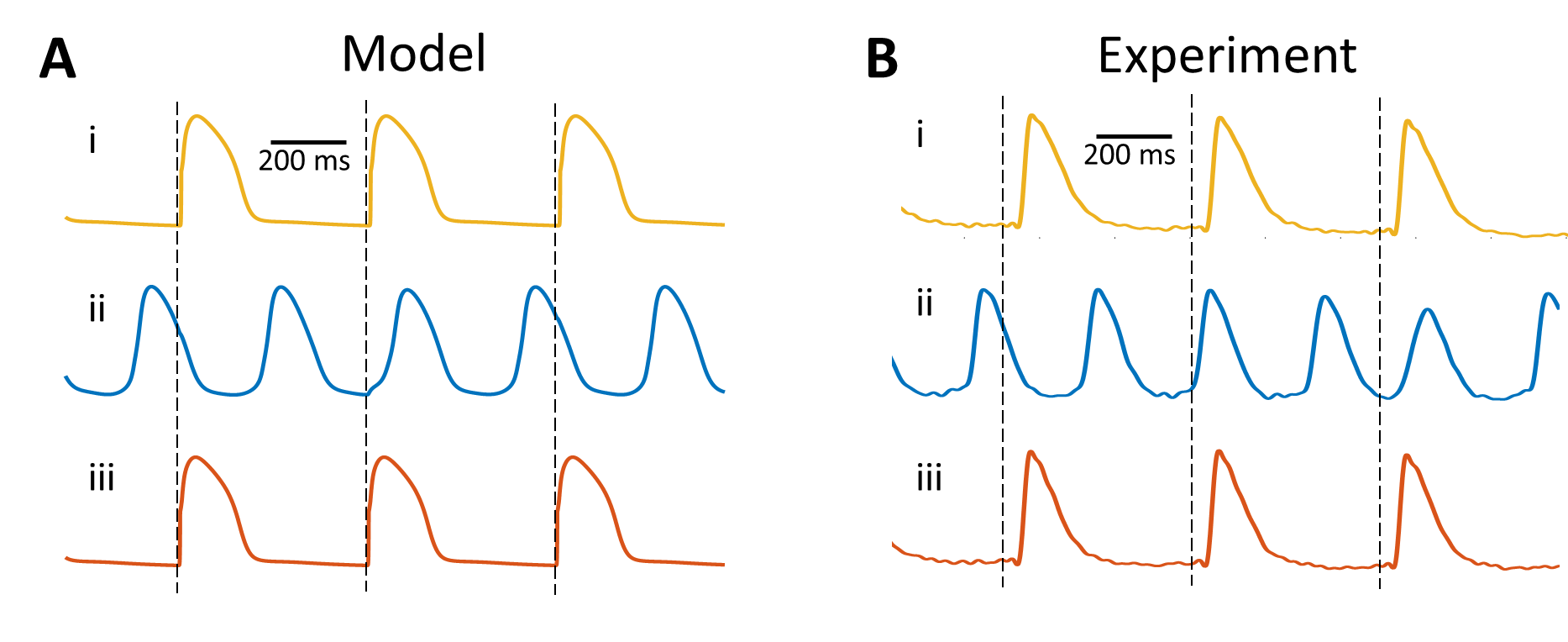


**Supplementary Figure 7.** *Comparison of modelled and experimental traces*

Modelled (**A**) and experimentally measured (**B**, reproduced from Fig. 2A) normalized voltage traces of a co-culture of ChR2-transduced MFBs (ChR2-MFBs) with CMs before (i, gold), during (ii, blue), and after (iii, orange) application of 10*I_0_ blue light to activate ChR2 current in MFBs. Vertical dashed lines show time of pacing.


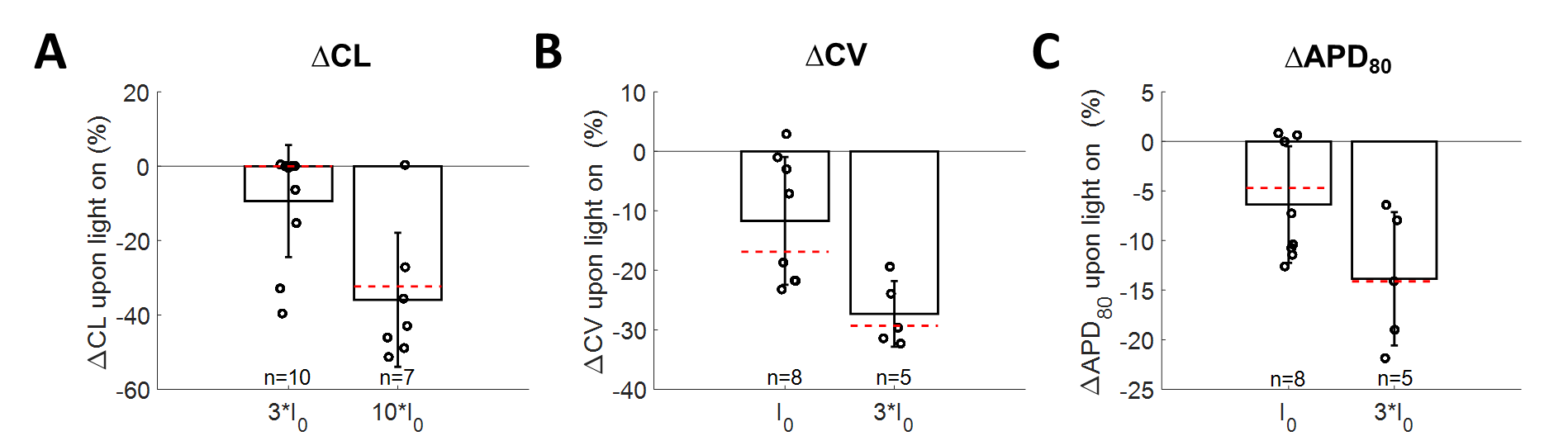


**Supplementary Figure 8.** *Comparison between experimental results and model.* Experimentally measured percent change in parameters in response to different light levels for beating cycle length (**A**), conduction velocity (**B**), and action potential duration (**C**) (adapted from Figs. 2B, 3B, and 4B). Dashed red lines indicate modelled values.


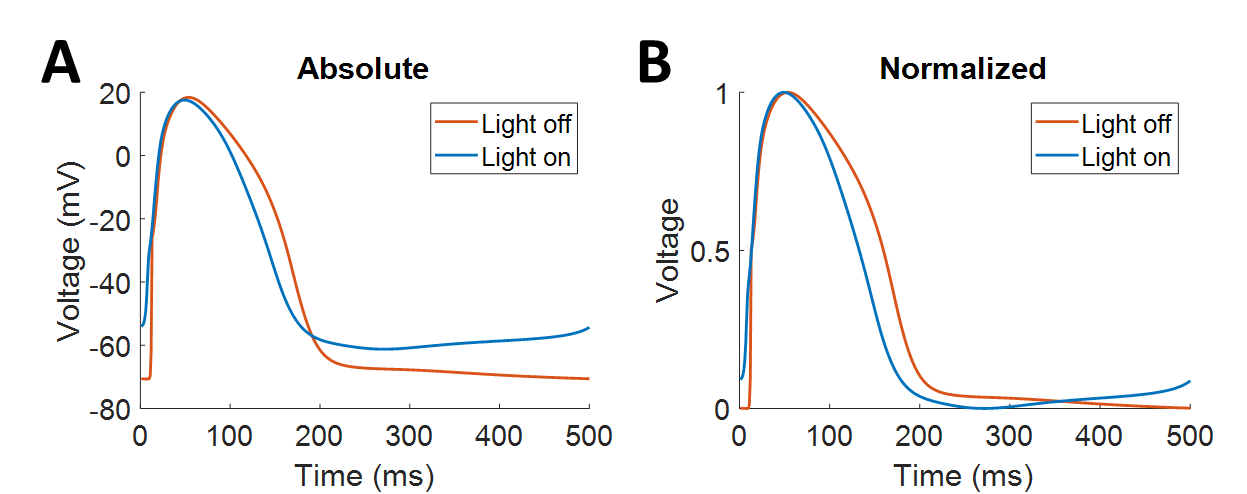


**Supplementary Figure 9.** *Amplitude changes contribute to changes in action potential duration.*

**A.** Modelled AP traces without (orange) and with (blue) 3*I_0_ light.**B.** Same as A, but with normalized AP traces, as are measured during optical mapping.


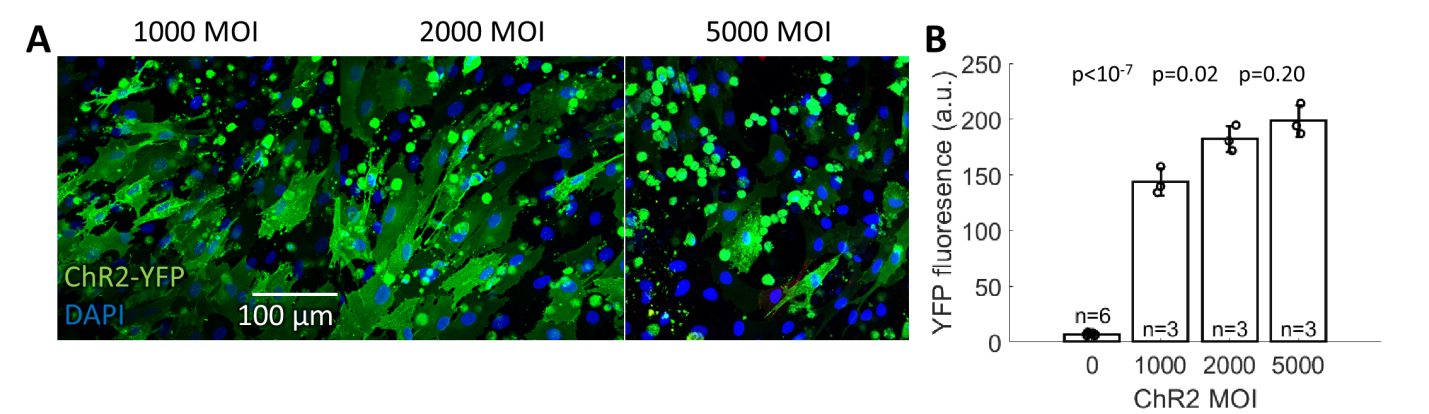


**Supplementary Figure 10.** *Transduction of myofibroblasts with ChR2.*

**A.** Confocal images of MFBs transduced with varying multiplicity of infection (MOI) of ChR2-YFP adenovirus. Green shows YFP, which marks cells transduced with ChR2, and blue shows DAPI (cell nuclei). All images are taken at the same scale and settings. **B.** YFP fluorescence, as measured by a fluorescence plate reader, was significantly increased by transducing with 2000 MOI versus 1000 MOI, while 5000 MOI only marginally increased YFP fluorescence. Therefore, MFBs were transduced in this study at approximately 2000 MOI.

**Supplementary Figure 11.** *Original and modified $\tau_{n_{Ks}}$ as a function of voltage.*

The I_Ks_ time constant was changed to the standard form $\tau_{n_{Ks}}=\frac{1}{\alpha_{n}+\beta_{n}}$, with α_n_ and β_n_ changed so the maximum rates matched those found in guinea pig ventricle, instead of being set to a constant as done in the original Korhonen model.


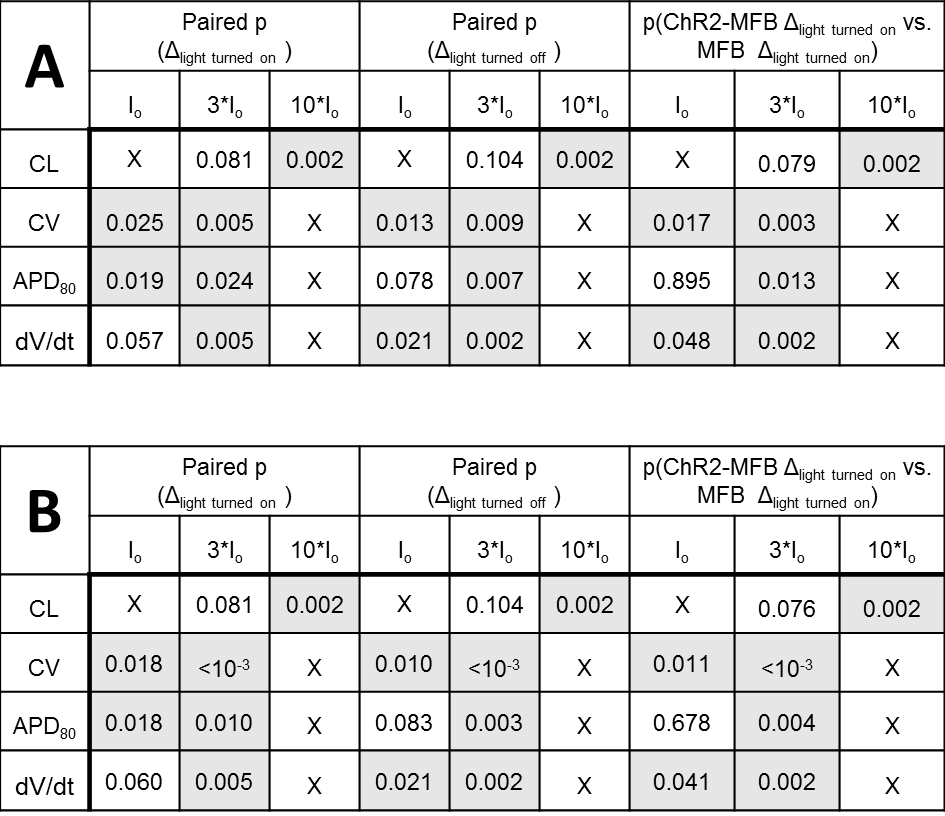


**Supplementary Table 1.** *Additional statistics for experimental data.*

P-values for absolute (**A**) and relative (**B**) changes of measured parameters at different light levels. The third column shows p-values comparing the response to light of ChR2-transduced MFBs and non-transduced MFBs, which were used for significance shown in Figs. 2-4. At I_0_, CL was always at the paced 500 ms, and at 10*I_0_, co-cultures almost always beat spontaneously, so these p-values are not calculated. p < 0.05 is shaded.


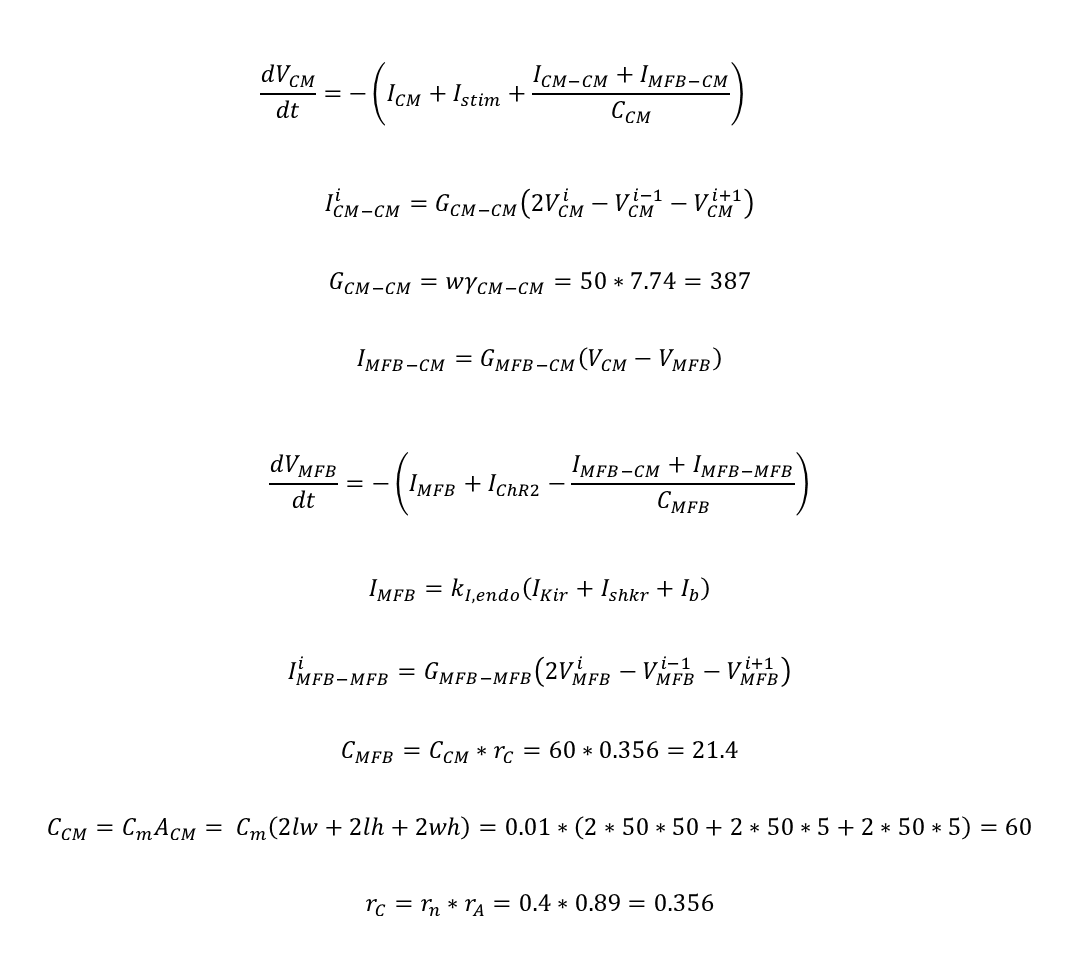


**Supplementary Table 2.** *Model equations for a 1-D cable of CM-MFB pairs.*

Sarcolemmal currents are normalized to cell capacitance, intercellular currents are not. Unit basis is ms, mV, pA, pF, µm, and nS. Superscript *i* denotes which cell (i.e. 1-30) the variable refers to. While all variables may have a different value for each cell, the equations for each cell are the same, so the superscript is omitted for simplicity, except in the equations for $I_{CM-CM}^{i}$ and $I_{MFB-MFB}^{i}$, in which neighboring cells in the cable interact.


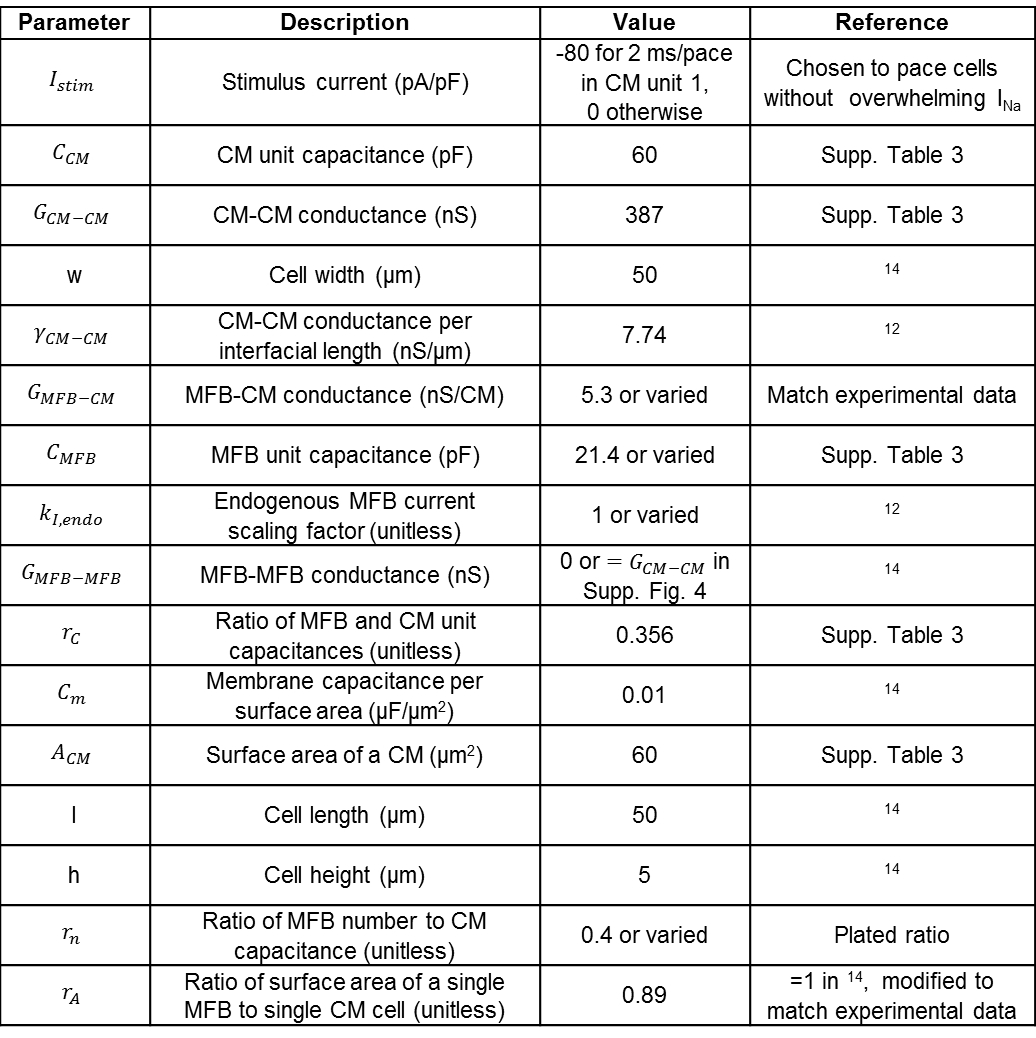


**Supplementary Table 3.** *Model parameters from Supplementary Table 3.*


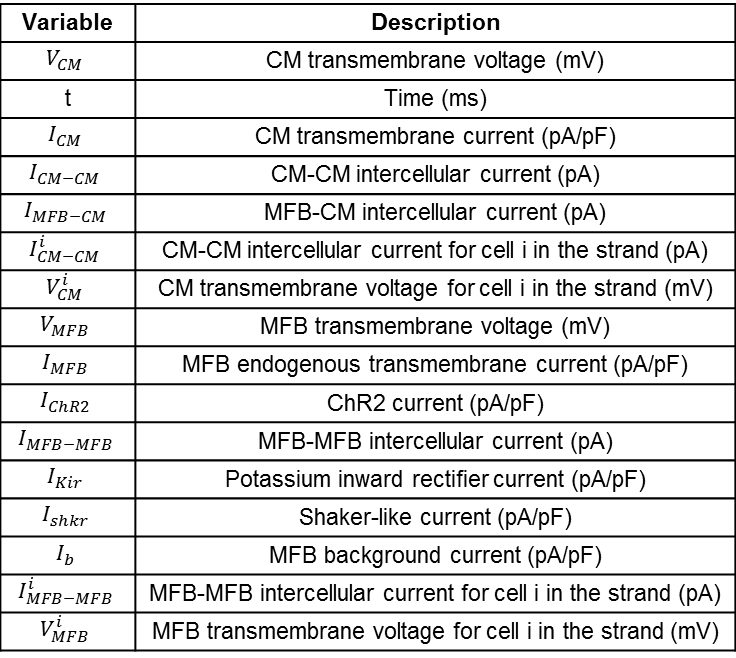


**Supplementary Table 4.** *Model variable definitions from Supplementary Table 3.*


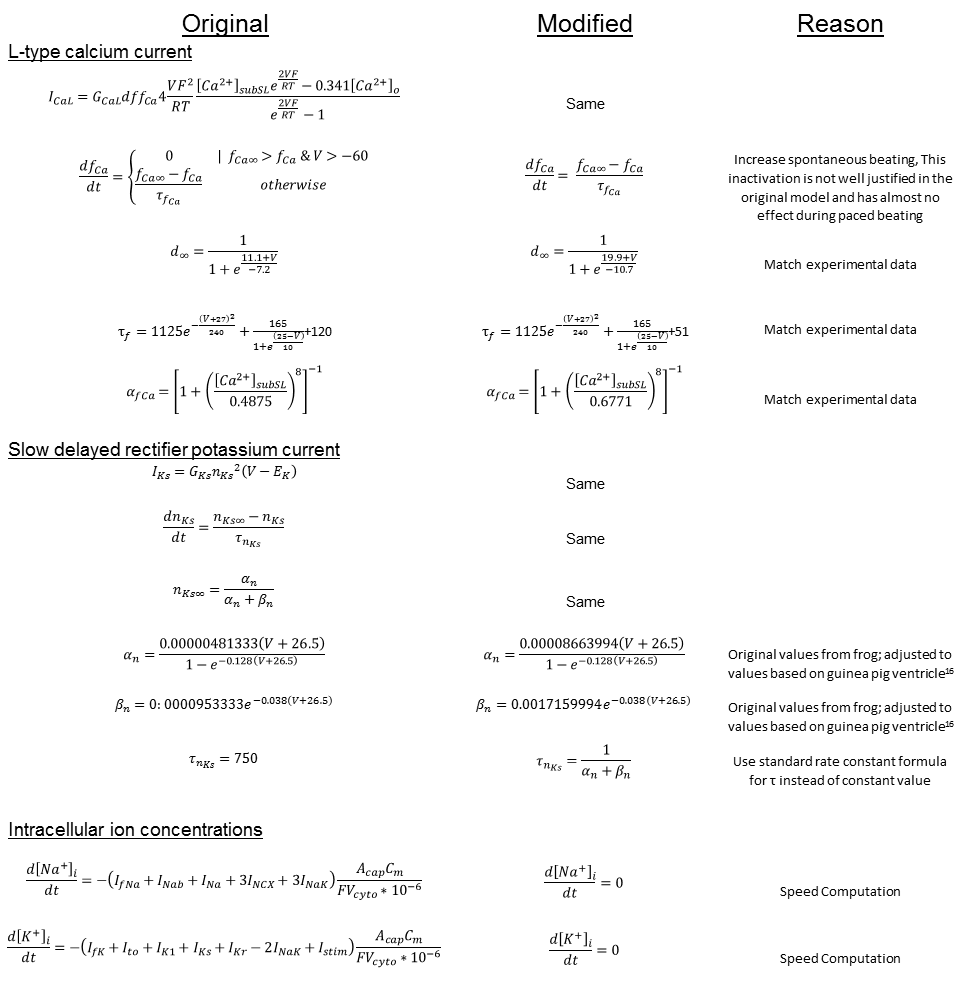


**Supplementary Table 5.** *Original and modified ion channel equations from Korhonen neonatal rat cardiomyocyte model.*

For simplicity, *V_CM_* is written as *V* in this table. Unit basis is ms, mV, pA, pF, µm, and nS.


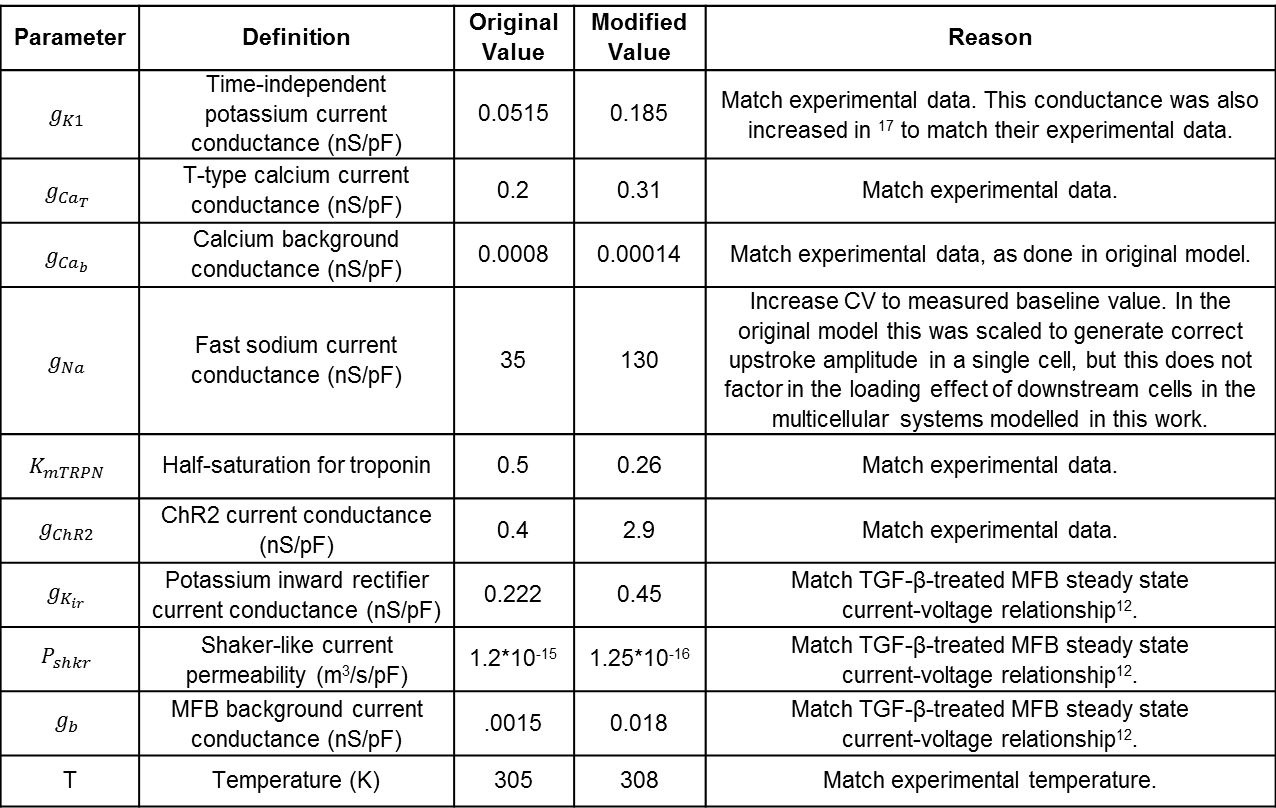


**Supplementary Table 6.** *Original and modified model parameters.*


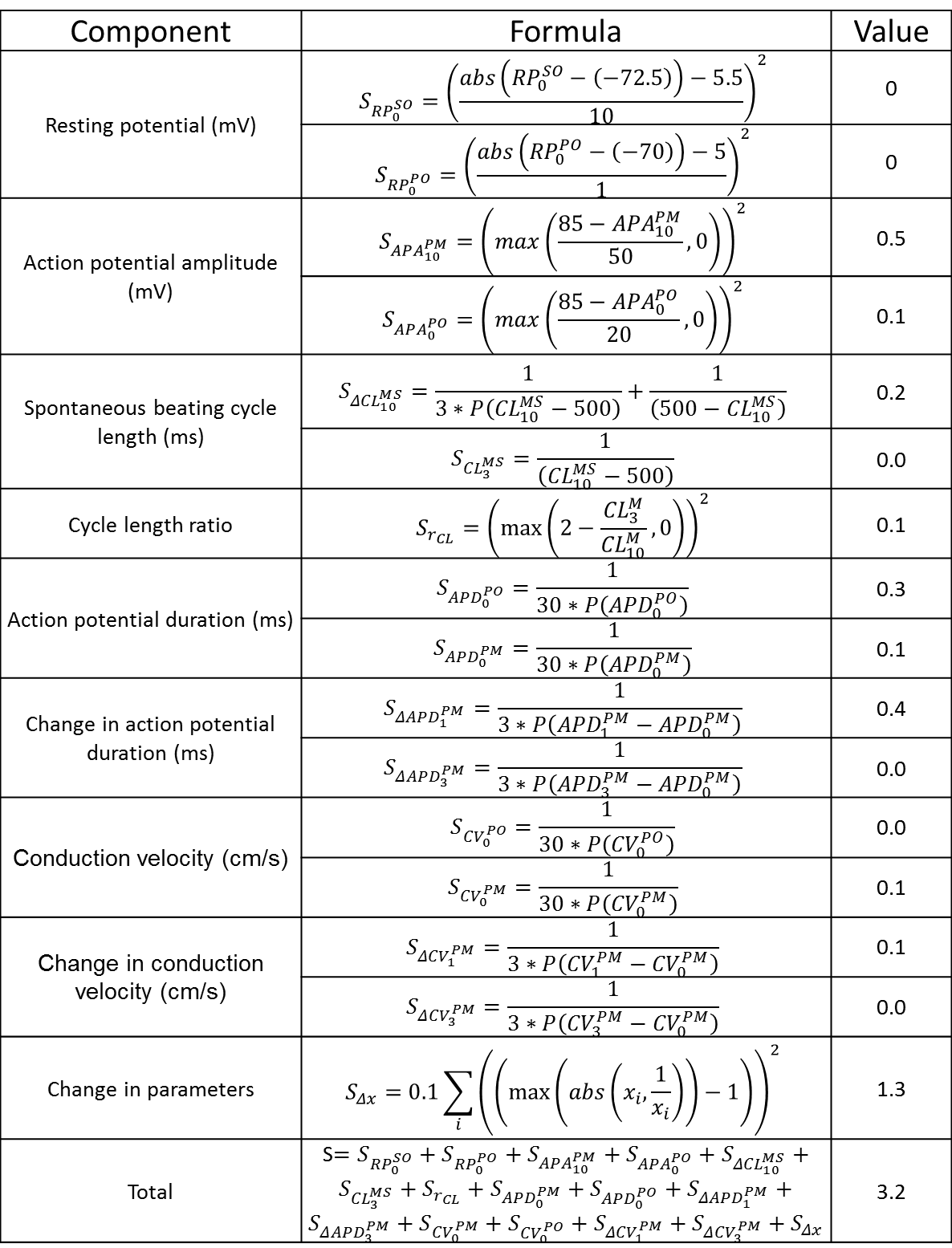


**Supplementary Table 7.** *Optimization scoring function.* Model variables were varied to minimize a scoring function to better match experimental data. APA is action potential amplitude. *x* is the factor by which a parameter is multiplied. Parameter subscript indicates light level (*I_0_), while superscript indicates pacing (P) or spontaneous beating (S), as well as absence (O) or presence (M) of ChR2-MFBs. *P(x)* is the probability of the value coming from the t-distribution of the corresponding experimental data.


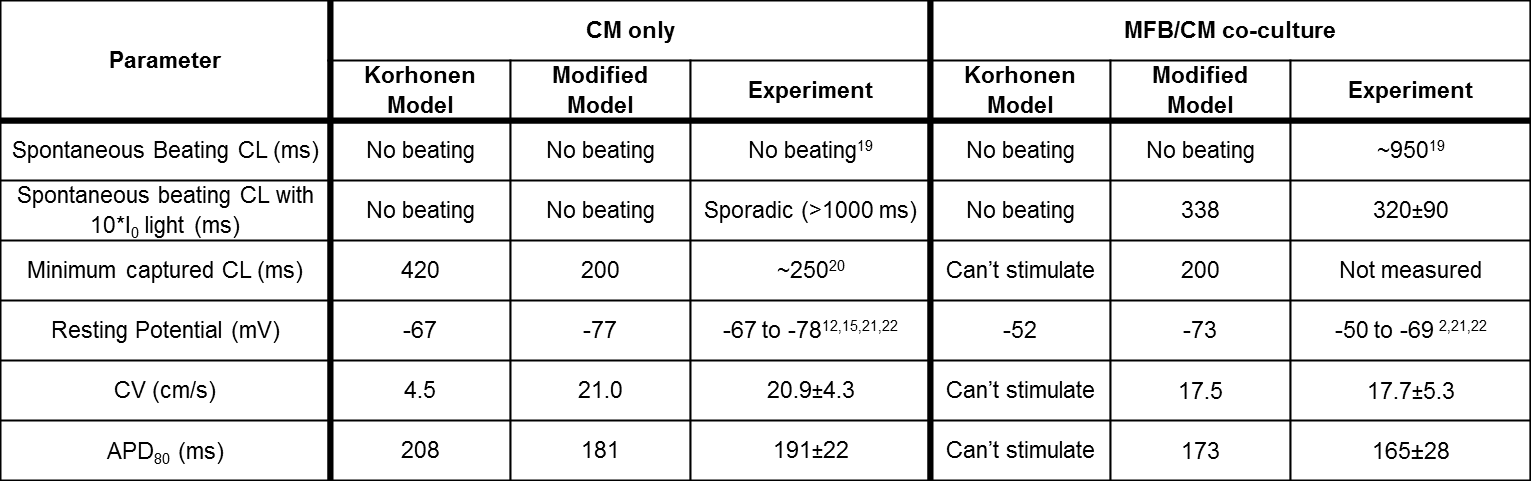


**Supplementary Table 8.** *Functional properties of original and modified CM model in a 1-D cable.*^192012,21,22^
